# Thermostable Branched-Chain Amino Acid Transaminases From the Archaea *Geoglobus acetivorans* and *Archaeoglobus fulgidus*: Biochemical and Structural Characterization

**DOI:** 10.3389/fbioe.2019.00007

**Published:** 2019-01-24

**Authors:** Michail N. Isupov, Konstantin M. Boyko, Jan-Moritz Sutter, Paul James, Christopher Sayer, Marcel Schmidt, Peter Schönheit, Alena Yu. Nikolaeva, Tatiana N. Stekhanova, Andrey V. Mardanov, Nikolai V. Ravin, Ekaterina Yu. Bezsudnova, Vladimir O. Popov, Jennifer A. Littlechild

**Affiliations:** ^1^Henry Wellcome Building for Biocatalysis, Biosciences, University of Exeter, Exeter, United Kingdom; ^2^Research Center of Biotechnology of the Russian Academy of Sciences, Moscow, Russia; ^3^Institut für Allgemeine Mikrobiologie, Christian-Albrechts-Universität Kiel, Kiel, Germany

**Keywords:** thermophilic archaea, branched-chain aminotransferases, substrate specificity, X-ray structural analysis, biocatalysis

## Abstract

Two new thermophilic branched chain amino acid transaminases have been identified within the genomes of different hyper-thermophilic archaea, *Geoglobus acetivorans*, and *Archaeoglobus fulgidus*. These enzymes belong to the class IV of transaminases as defined by their structural fold. The enzymes have been cloned and over-expressed in *Escherichia coli* and the recombinant enzymes have been characterized both biochemically and structurally. Both enzymes showed high thermostability with optimal temperature for activity at 80 and 85°C, respectively. They retain good activity after exposure to 50% of the organic solvents, ethanol, methanol, DMSO and acetonitrile. The enzymes show a low activity to (*R*)-methylbenzylamine but no activity to (*S*)-methylbenzylamine. Both enzymes have been crystallized and their structures solved in the internal aldimine form, to 1.9 Å resolution for the *Geoglobus* enzyme and 2.0 Å for the *Archaeoglobus* enzyme. Also the *Geoglobus* enzyme structure has been determined in complex with the amino acceptor α-ketoglutarate and the *Archaeoglobus* enzyme in complex with the inhibitor gabaculine. These two complexes have helped to determine the conformation of the enzymes during enzymatic turnover and have increased understanding of their substrate specificity. A comparison has been made with another (*R*) selective class IV transaminase from the fungus *Nectria haematococca* which was previously studied in complex with gabaculine. The subtle structural differences between these enzymes has provided insight regarding their different substrate specificities.

## Introduction

Transaminases (TAms; aminotransferases; EC2.6.1.-) catalyse a transfer of an amino group between a donor substrate and an acceptor molecule (Mehta et al., 1993). They are involved in the metabolism of most natural amino acids and collectively have a broad substrate range. This makes them very useful for the biocatalytic synthesis of chiral amino acid analogs (Leuchtenberger et al., 2005; Tufvesson et al., 2011) such as *tert*-leucine (Taylor et al., 1998) and homophenylalanine (Cho et al., 2003).

The TAms belong to a large group of enzymes that utilize pyridoxal 5′-phosphate (PLP) (Hayashi, 1995; Jansonius, 1998). PLP is the biologically active form of vitamin B6 (Braunstein and Shemyakin, 1953; Metzler et al., 1954). The mechanism of these enzymes involves the PLP cofactor forming a covalent bond (a Schiff base) with the NZ atom of the active-site lysine (internal aldimine). In the first half of the TAm reaction, a Schiff base is formed between the donor substrate and PLP (external aldimine). After several reaction stages, the substrate amino group is transferred to the cofactor to form pyridoxamine 5′-phosphate (PMP) with the release of the ketone product. In the second half-reaction the PMP amino group is transferred to an acceptor ketone or aldehyde to form the PLP internal aldimine and a primary amine or a chiral amino product. Most TAms use an α-ketoglutarate (AKG) acceptor substrate to form a glutamate product. Some TAms, however utilize the pyruvate acceptor to form alanine and are usually referred to as substrate:pyruvate TAms (Ward and Wohlgemuth, 2010; Sayer et al., 2012).

The TAms are currently widely used in industrial biocatalysis for both the resolution and the asymmetric synthesis of chiral amines which are used as building blocks for drug intermediates. There is specific interest in TAms capable of the production of both (*S*) and (*R*)-chiral intermediates of non α-amino acid substrates. These enzymes catalyse amination of substrates with a ketone/aldehyde component distal to the carboxyl group [ω-TAms; (Malik et al., 2012)] or with no carboxyl group [amine TAms; (Shin et al., 2003)]. Several Pfam class III (*S*)-selective ωTAms (Punta et al., 2012) from *Vibrio fluvialis* (Shin et al., 2003), *Chromobacterium violaceum* and *Pseudomonas aeruginosa* (Kaulmann et al., 2007), *Arthrobacter* sp. and *Bacillus megaterium* (van Oosterwijk et al., 2016) have been biochemically and structurally characterized (Humble et al., 2012; Midelfort et al., 2013; Sayer et al., 2013). They show activity toward the (*S*)-isoform of α-methylbenzylamine [(*S*)-MBA] and also toward other amines and ω-amino acids such as β-alanine. The amino acceptor can be pyruvate and a variety of other ketones and aldehydes (Kaulmann et al., 2007).

Most α-TAms and (*S*)-ωTAms, are specific toward the (*S*)-enantiomer of the substrate and have a PLP type I fold. Enzymes of the Pfam TAm class IV have a PLP type IV fold and catalyse transamination of branched chain L-amino acids (BCAAs), D-amino acids or ω-amino acids. The catalysis in class IV TAms occurs on the *re*-side of the cofactor, which differs from most of the other PLP enzymes where the reactions occur on the *si*-side. Since both (*S*) and (*R*)-enantiomers can be turned over within the same PLP type IV scaffold, enzymes capable of transamination of (*R*)-ω-substituted amines are found within this class of TAms. By comparing the sequence for class IV enzymes which are potentially capable of (*R*)-ωTAm activity a sequence fingerprint has been established (Höhne et al., 2010). Biochemical characterization of a number of these enzymes has demonstrated a high enantioselectivity toward the substrates (*R*)-α-methylbenzylamine [(*R*)-MBA], (2*R*)-amino-4-phenylbutane and (2*R*)-aminohexane.

The production of the (*R*)-ω-substituted chiral amines is a challenging process using conventional chemical synthesis. Therefore, enzymes capable of catalyzing the selective transamination of such compounds have received significant interest. An example of such an enzyme is a mutant (*R*)-specific transaminase from *Arthrobacter* sp. (ArRMut11), created in a collaboration between Codexis and Merck, which was able to catalyse specific amination of the sterically demanding 1,3-ketoamides to generate the (*R*)-chiral amine for the manufacture of Sitagliptin, a treatment for type II diabetes (Savile et al., 2010). This mutant ArRMut11 enzyme has been reported in reactions for amination of bicyclic ketones including tetralone (Mutti et al., 2011; Pressnitz et al., 2013). The crystal structures have been reported for several class IV TAm enzymes including the fungal (*R*)-amine:pyruvate ωTAms from *Aspergillus fumigatus* (Thomsen et al., 2014), *Aspergillus terreus* (Łyskowski et al., 2014) and *Nectria haematococca* (Sayer et al., 2014), and the bacterial (*R*)-selective ωTAm ArRMut11 from *Arthrobacter* sp (Guan et al., 2015).

There is an increasing demand for enzymes which are more robust to the demanding conditions used in industry. Enzymes found in thermophilic organisms have increased thermostability and are more tolerant to organic solvents and proteolytic cleavage. Solvent stability is advantageous since non-natural substrates used industrially often require the addition of organic solvents to the reaction mixture for substrate solubilisation (Littlechild et al., 2007). Also the biocatalytic process can be carried out at elevated temperatures where many non-natural substrates have improved solubility when using a thermostable enzyme which can be reused through several reaction cycles. This reduces the overall cost of the enzyme in the industrial process which is often a limitation in the development of a biocatalytic process.

**Scheme 1:**
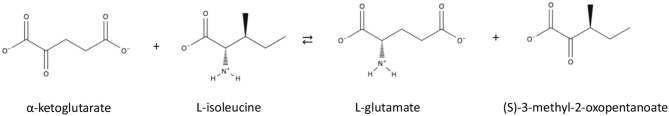
Reaction of Branched Chain TAms.

Branched chain TAms (BCATs) catalyse reversible transamination of branched chain amino acids (shown in Scheme 1). Recently, archaeal thermophilic BCATs have been biochemically characterized from *Thermococcus* sp (Uchida et al., 2014) and biochemically and structurally studied from the thermophile *Thermoproteus uzoniensis* (Boyko et al., 2016).

This paper reports the identification, biochemical and structural characterization of two new thermostable archaeal class IV TAms from *Geoglobus acetivorans* (Querellou et al., 2009) and *Archaeoglobus fulgidus* (Stetter, 1988). Both of these hyper-thermophilic archaea have been isolated from different deep sea hydrothermal vents and they share 79.2% sequence identity. The structures of the *G. acetivorans* enzyme have been determined in the internal aldimine form and in complex with the amino acceptor AKG and the structures of the *A. fulgidus* enzyme in the internal aldimine form and in complex with the inhibitor gabaculine. The different structural complexes of these related enzymes have given further insight into the overall mechanism of BCATs and their high stability for industrial application and their substrate specificity.

## Results and Discussion

### Enzyme Cloning, Expression, and Purification

The genes encoding two putative BCATs were identified in the genomes of *G. acetivorans* (Mardanov et al., 2011) and *A. fulgidus* (Klenk et al., 1997). Both proteins called GEO1900 and AF0933 have been cloned and over-expressed in a soluble form in *Escherichia coli* and have been purified to homogeneity using metal affinity and size exclusion chromatography. The recombinant BCATs GEO1900 (MW of subunit 32.6 kDa, 292 amino acids) and AF0933 (MW of subunit 32.4 kDa, 290 amino acids) are closely related with a sequence identity of 79.2% and 94.8% similarity. When purified by high resolution gel filtration chromatography the native molecular weight of the two enzymes varied with the GEO1900 approximately 70 kDa, indicating that the enzyme was a homodimer with small amount of tetramer in solution (Figure S1). However, the AF0933 enzyme had a native molecular mass of approximately 220 kDa as determined by size exclusion chromatography, indicating that it forms a homo-hexamer with only small amounts of a homo-dimeric enzyme observed (Figure S2). Both proteins showed absorption at 420 nm indicating that the cofactor PLP was bound in the aldimine form (data not shown). Both of the GEO1900 and AF0933 proteins run as a single band of 36 kDa on SDS-PAGE which is in line with the calculated molecular mass of the His-tagged proteins (35.4 kDa).

### Biochemical Characterization of GEO1900 and AF0933

Both enzymes, GEO1900 and AF0933, showed significant activity toward keto analogs of BCAAs (Tables 1, 2). The apparent kinetic parameters for the AF0933 catalyzed transamination reaction between L-glutamate and the branched chain oxoacids were similar with a preference for 3-methyl-2-oxopentanoate. The extrapolated *V*_max_ and *K*_m_ values for L-glutamate were significantly higher (Table 2 and Figure S7). These values of *V*_max_ and *K*_m_ point to the low affinity binding of L-Glu in the active site. This was observed earlier for other BCATs from *P. aeruginosa* and *Gluconobacter oxydans* (Norton and Sokatch, 1970; Tachiki and Tochikura, 1973; Kanda et al., 1995). For GEO1900 the extrapolated V_max_ and K_m_ values for pyruvate and AKG were (1.9 ± 0.1 U/mg and 10.6 ± 1.9 mM) and (18.2 ±1.2 U/mg and 1.9 ± 0.6 mM), respectively. The calculated catalytic efficiency constant for GEO1900 toward AKG was found to be higher than toward pyruvate, 9.8 s^−1^ mM^−1^ compared to 0.1 s^−1^ mM^−1^. Both values fall within the range of the calculated values for the canonical BCATs (Bezsudnova et al., 2017).

**Table 1:** The specific activity of GEO1900 and AF0933 toward different keto acids.

**Amino acceptor**	**Specific activity (U/mg)**	
	**GEO1900^a^**	**AF0933^b^**
3-methyl-2-oxobutyrate	n.a.	5.14
4-methyl-2-oxopentanoate	7.7	3.05
3-methyl-2-oxopentanoate	7.1	3.23
2-oxopentanoate	n.a.	6.51
2-oxohexanoate	n.a.	4.26
2-oxooctanoate	n.a.	2.4
2-oxobutyrate	1.3	n.a.
pyruvate	0.56	0.73
phenylglyoxylate	n.a.	4.55
β-phenylpyruvate	n.a.	2.84
indole-3-pyruvate	n.a.	1.02

a *5 mM L-glutamate was used as the amino donor and 5 mM keto acid was used at 65°C in 50 mM phosphate buffer, pH 8.0; supplemented with 60 μM PLP*.

b *10 mM L-glutamate was used as the amino donor and 10 mM keto acid was used at 70°C in 100 mM Tris-HCl buffer pH 7.5 supplemented with 0.1 mM PLP*.

*Not all donor acceptor pairs were tested for both enzymes*.

**Table 2:** The apparent kinetic parameters of the transamination reaction catalyzed by AF0933.

**Substrate**	**V_**max**_ (U/mg)**	**K_**m**_ (mM)**	**k_**cat**_/K_**m**_ (s^**−1**^ mM^**−1**^)**
3-methyl-2-oxobutyrate^a^	6.3	0.42	8.85
4-methyl-2-oxopentanoate^a^	5.5	0.25	12.98
3-methyl-2-oxopentanoate^a^	3.9	0.13	17.7
L-glutamate^b^	64.6^*^	121	0.32

a *At 10 mM L-glutamate*.

b *At 5 mM 3-methyl-2-oxobutyrate*.

* *The V_max_ for L-glutamate is an extrapolated value calculated at saturated concentration of co-substrate*.

As for the activity toward amino donors the profile of activity toward amino acids for GEO1900 (Table S1) was similar to that reported for related BCATs from *E.coli, Bacillus brevis* and *Pseudomonas aeruginosa* (Norton and Sokatch, 1970; Lee-Peng et al., 1979; Inoue et al., 1988; Kanda et al., 1995; Yu et al., 2014). GEO1900 was highly active toward branched-chain amino acids and their isomers. The AF0933 catalyzed the transamination between L-valine and AKG with a specific activity of 5.76 U/mg and between L-alanine and 3-methyl-2-oxobutanoic acid with a lower specific activity of 0.16 U/mg. When the enantio-preference of the GEO1900 and AF0933 in the reaction with the (*R*) and (*S*)-isomers of methylbenzylamine (MBA) was investigated using the acetophenone assay they both showed a small but significant activity toward (*R*)-MBA at pH 7.5, with a value of 2 and 10 mU/mg with 2-oxobutyrate as the keto substrate, respectively. Neither BCAT showed any activity toward (*S*)-MBA.

The temperature optimum for the transamination reaction catalyzed by GEO1900 and AF0933 was found to be 80 and 85°C, respectively (Figures S3, S5). The pH optimum for amination of keto acids with L-Glu was between pH 7.0 and 8.0 for both enzymes. The thermostability of GEO1900 and AF0933 was evaluated using the analysis of the residual activity after pre-incubation of the enzyme for 2 h at different temperatures. Significant loss of activity of GEO1900 was observed at temperatures higher than 50°C. The pre-incubation of the enzyme at 70°C resulted in 25% loss of activity after 2 h (Figure S4). At the same time more than 60% of the AF0933 residual activity remained after pre-incubation at 75°C for 2 h (Figure S6). The pre-incubation of AF0933 at 85°C for 60 min resulted in a drop of the residual activity to 30%.

The solvent stability of GEO1900 and AF0933 was tested in ethanol, methanol, DMSO and acetonitrile. After 1 h of incubation in up to 50% of these solvents GEO1900 retained close to 100% activity at both 20°C and 55°C. For AF0933 after 1 h incubation of the enzyme in up to 50% of the tested solvents at the similar temperatures 80% of the original activity remained.

### Structural Studies

**Figure 1:**
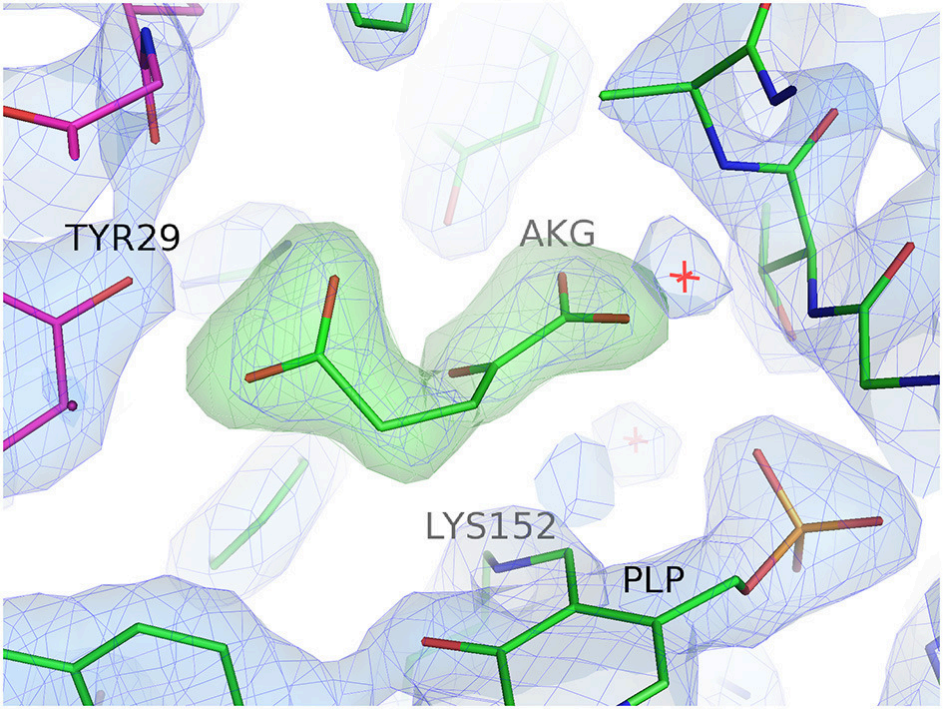
Electron density for the bound substrate AKG in the active site of subunit A of the GEO1900. The 2Fo-Fc electron density map (blue) is contoured at 1.5σ, the positive (green) and negative (red) Fo-Fc electron density maps are contoured at 3.5σ and −3.5σ, respectively. Figure drawn using PyMOL (Schrödinger, LLC).

The GEO1900 holoform (internal aldimine form; GEO1900_holo) and the unproductive complex of holoenzyme with the keto substrate AKG-(GEO1900_AKG) have been determined to 1.9 and 2.2 Å resolution, respectively, in the trigonal space group P3_2_21. There are three GEO1900 subunits per asymmetric unit, which form a tight hexamer with 32 point group symmetry with their symmetry mates. The AF0933 structure has been determined in the holoform (AF0933_holo) and in complex with the inhibitor gabaculine (GABC) (AF0933_GABC) to 2.1 and 2.0 Å resolution, respectively, in the orthorhombic P2_1_2_1_2_1_ space group. The asymmetric unit contains a hexameric AF0933 molecule which is very similar to GEO1900 hexamer observed in the crystal. All of the structures have been refined to low R-factors with good stereochemical parameters as shown in Table 3. In the GEO1900 structures two N-terminal amino acids which are not present in AF0933, were not defined in the electron density. The interdomain loop residues 121–129 could be fully built into the subunits A and B in GEO1900_AKG structure. The bound ligand AKG was found only in subunit A and was clearly defined in the electron density (Figure 1). The interdomain loop could only be fully built in one subunit (D) out of the six subunits in both the AF0933_holo and AF0933_GABC structures. The crystal contacts in AF0933 appear to be contributing to the lower disorder of the loop in the D subunit. The inhibitor, gabaculine was not present in the electron density in the subunit F of AF0933, therefore this subunit was built as an internal aldimine in the AF0933_GABC structure. In other subunits the gabaculine-PLP adduct was modeled with partial occupancy in the range of 0.6–0.7. There are no residues in a *cis* conformation in the well-defined regions of the GEO1900 and AF0933 structures. Residue Glu249 is a Ramachandran plot outlier (Ramakrishnan and Ramachandran, 1965) in all subunits of both AF0933 structures. The corresponding Glu251 has similar main chain angles in the GEO1900 structures, but lies in a generously allowed region of the Ramachandran plot (Laskowski et al., 1993). The fungal class IV TAms from *Aspergillus* and *Nectria* species (Łyskowski et al., 2014; Sayer et al., 2014; Thomsen et al., 2014) have a Gly residue at this position. Many residues in both of the GEO1900 and AF0933 structures have been modeled with multiple conformations of some amino acid side chains. The residues Gln107 and Asn108 were modeled with alternative conformations of the main chain in most subunits of the AF0933_holo structure and in the subunits E and F of the AF0933_GABC structure.

**Table 3:** Summary of data processing and refinement statistics.

	**Holo enzyme GEO1900**	**α-ketoglutarate complex GEO1900**	**Holo enzyme AF0933**	**Gabaculine complex AF0933**
Diffraction source	BL41XU, Spring8	BL41XU, Spring8	I04-1, Diamond	I04-1, Diamond
Wavelength (Å)	1.0	1.0	0.92	0.92
Space group	P3_2_21	P3_2_21	P2_1_2_1_2_1_	P2_1_2_1_2_1_
*a, b, c* (Å)	117.65, 117.65, 135.98	117.31, 117.31, 135.31	72.92, 139.12, 167.57	73.21, 140.23, 168.32
α, β, γ (°)	90, 90, 120	90, 90, 120	90, 90, 90	90, 90, 90
Resolution range (Å)	58.83–1.90 (2.00–1.90)^a^	67.66–2.20 (2.26–2.20)	69.60–2.10 (2.14–2.10)	67.17–1.98 (2.01–1.98)
Number of unique reflections	85638 (11949)	55018 (4457)	99985 (4906)	119864 (5675)
Completeness (%)	99.6 (98.6)	99.9 (99.8)	99.9 (99.6)	99.0 (95.6)
Average redundancy	19.8 (19.3)	17.7 (18.4)	6.3 (5.4)	5.6 (4.7)
<*I*/σ(*I*)>	31.75 (4.0)	15.1 (4.9)	10.0 (0.8)	11.2 (0.8)
R_meas_ (%)^b^	6.8 (79.2)	13.3 (67.4)	14.7 (251.1)	12.8 (233.5)
CC_1/2_ (%; Diederichs and Karplus, 1997)	100 (92.9)	99.8 (92.6)	99.7 (29.2)	99.7 (24.9)
Overall *B* factor from Wilson plot (Å^2^)^c^	39.8	42.3	51.3	44.8
*R_*fact*_* (%)	15.3	15.0	18.3	18.1
*R*_free_ (%)	18.8	20.8	21.8	20.9
Refined protein atoms	6671	6836	14432	14440
Refined solvent atoms	626	508	754	828
Refined ligand atoms	0	10	0	125
**AVERAGE B FACTOR (Å**^**2**^**)**				
Protein	37.9	38.2	45.8	42.7
Solvent	45.3	40.9	50.6	47.9
R.m.s.d. bond lengths (Å)	0.021	0.019	0.013	0.013
R.m.s.d. bond angles (°)	1.99	2.11	1.78	1.69
**RAMACHANDRAN PLOT ANALYSIS, RESIDUES IN (%)^d^**				
Most favored regions	90.5	89.9	90.6	90.7
Additional allowed regions	8.7	8.8	8.6	8.5
Generously allowed regions	0.8	1.1	0.4	0.4
Disallowed regions	0	0.3	0.4	0.4

a *Values for the highest resolution shell are given in parentheses*.

b *meas **=** Σ_h_[m/(m – 1)]^1/2^ Σ_i_|I_h, i_| – < I_h_>/ Σ_h_ Σ_i_I_h, i_ (Karplus and Diederichs, 2012)*.

c *Wilson B-factor was estimated by SFCHECK (Vaguine et al., 1999)*.

d *Ramachandran plot analysis was performed by PROCHECK (Laskowski et al., 1993)*.

### Subunit Structure

**Figure 2:**
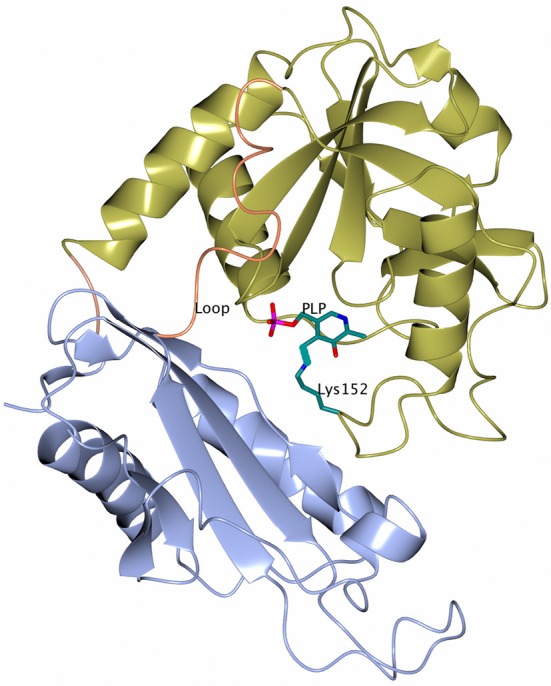
A cartoon representation of the subunit of GEO1900 with the large domain shown in gold and the small domain shown in ice blue. The loops connecting the domains are shown in coral. The cofactor PLP and the active site lysine 152 are shown as stick models with carbon atoms shown in green. Figures 2–5, 7, 8 are drawn using CCP4mg (McNicholas et al., 2011).

The subunit of the archaeal BCATs has a PLP type IV fold typical of other BCATs, D-amino acid TAms and (*R*)-selective ωTAms (Mehta et al., 1993; Höhne et al., 2010) and consists of two α/β domains, a small domain and a large domain connected by two inter-domain loops (Peisach et al., 1998; Okada et al., 2001; Goto et al., 2003; Hirotsu et al., 2005; Boyko et al., 2016) (Figure 2).

The small domain (residues 1–119 and 287–292 in GEO1900) is composed of three α-helices wrapped around a seven stranded antiparallel β-sheet with topology −1, +4x, +1, −2x, −1, +4x as defined by PROMOTIF (Richardson, 1981; Kabsch and Sander, 1983; Hutchinson and Thornton, 1996) with the C-terminus contributing to the last β-strand. The large PLP-binding domain (residues 130–285) has six α-helices surrounding an eight stranded β-sheet with topology −1x, −1, +5, +1, +1, −3, −1 and direction − + + + − + −. Both domains are connected via a short link at C-terminus (residues 286–287) and a long inter-domain loop (residues 120–129).

### Quaternary Structure of BCATs

**Figure 3:**
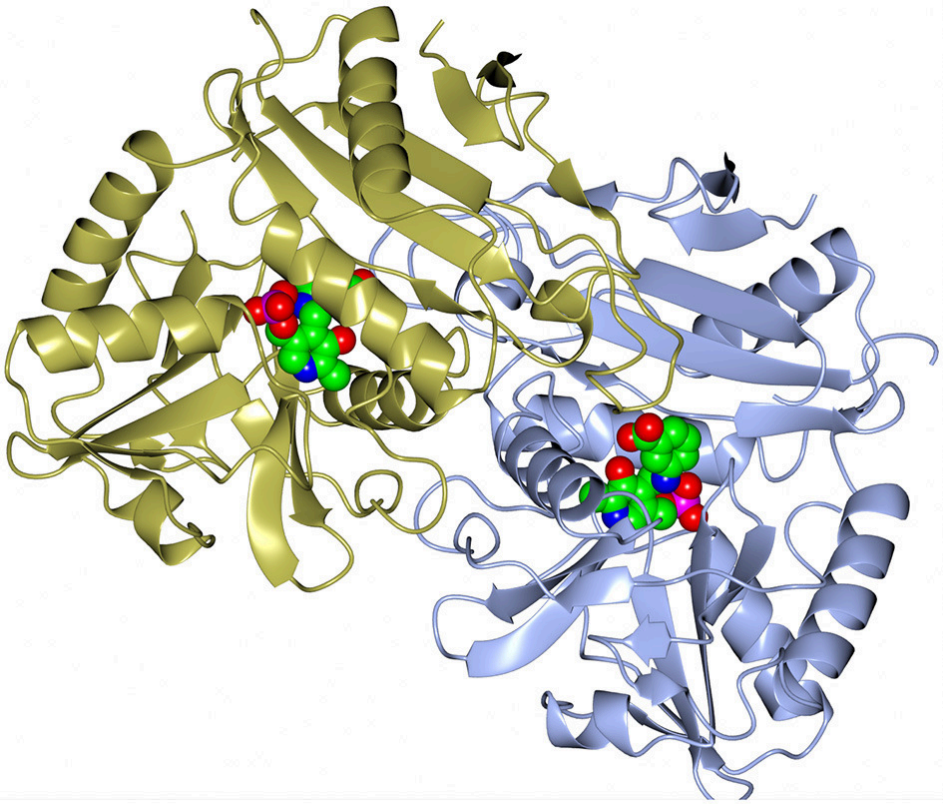
A cartoon representation of the dimer of the AF0933 BCAT gabaculine complex. The gabaculine-PLP covalent adduct molecules are shown as spheres.

**Figure 4:**
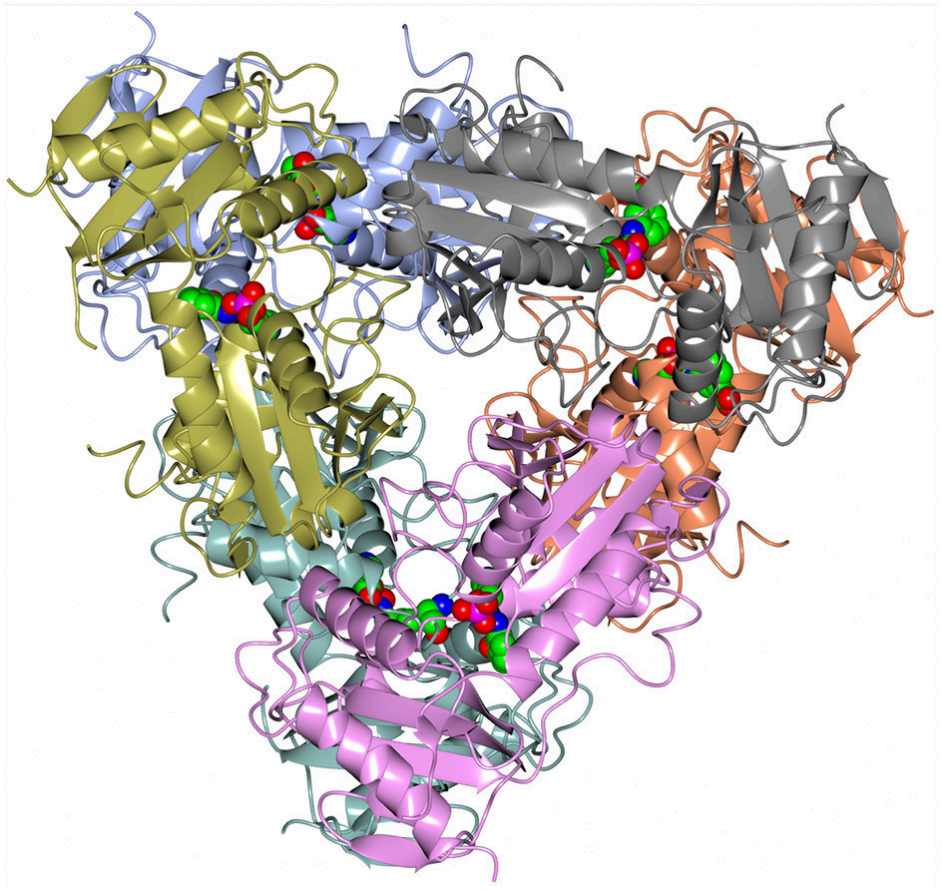
A cartoon representation of the AF0933 BCAT holo structure in the native hexameric form. The PLP cofactor molecules are shown as spheres.

A dimer formation is required for the BCAT catalytic activity, since residues from both subunits in the dimer contribute to the active site (Figure 3). However, both GEO1900 and AF0933 form hexamers (trimers of dimers) in the crystal (Figure 4), with the GEO1900 hexamer located on a crystallographic dyad. Dimerization of GEO1900 results in 2400 Å^2^ or 18% of each subunit solvent accessible surface area being buried (Krissinel and Henrick, 2007). Hexamer formation of GEO1900 in the crystal leads to an additional 14% of each subunit surface area being buried. Similarly, dimerization of AF0933 buries 19% of subunit surface area with additional 14% buried upon hexamer formation. This is very much in line with other bacterial BCATs that form a hexameric structure in the crystal (Okada et al., 2001; Chen et al., 2012; Boyko et al., 2016), with organization of all hexamers being very conserved, the core rmsd in Cα positions are comparable for both dimers and hexamers in pairwise alignment. Interestingly, known structures of other members of group IV proteins—D-amino acid TAms, bacterial and fungal amine TAms are homodimers (Peisach et al., 1998; Sayer et al., 2014; Thomsen et al., 2014). In this context a dimeric structure in solution observed by size exclusion chromatography for GEO1900 and *T. uzoniensis* BCAT (Boyko et al., 2016), and a small dimeric fraction for AF0933 suggests an equilibrium between hexameric and dimeric forms, since only dimer is required for catalysis. The AF0933 is predominantly hexameric in solution (Figure S2) which may account for its increased thermal optimum over that of GEO1900.

### Active Site

The active site of the archaeal BCATs is located at the bottom of the cleft formed by residues of both domains of one subunit as well as of the small domain of an adjacent subunit within the catalytic dimer. The same arrangement has also been observed in other BCATs (Okada et al., 2001) and generally in all enzymes with a PLP type IV fold (Peisach et al., 1998; Sayer et al., 2014; Thomsen et al., 2014). A PLP molecule is covalently bound to the catalytic Lys152 in the GEO1900 active site (Lys150 in AF0933). Since most active site residues are conserved between the two BCATs the GEO1900 numbering will be used henceforth.

In the holo and complex structures of the archaeal BCATs the PLP moiety is clearly visible in the electron density map and has B factors similar to those of the neighboring protein residues, indicating a full occupancy of the cofactor. The PLP forms a number of contacts with the protein molecule as found in other BCATs (Hutson, 2001; Okada et al., 2001; Yennawar et al., 2001). Apart from the Schiff base formed by the NZ of Lys152 residue, the pyridine ring of the cofactor forms hydrogen bonds to the conserved Tyr156 and Glu185 residues. The PLP phosphate group forms a salt bridge with Arg53 and a number of hydrogen bonds to the main chain nitrogens and side chains of residues Thr212 and Thr248, and the main chain nitrogen of Ile211, and two water molecules (Figure S8). The active site of BCAT can be subdivided into two pockets, A and B. In GEO1900, the B-pocket is formed by the residues Gly36, Arg38, Tyr89, Gly188, Thr248, and Ala249 (Figure S8). When compared to the structure of *E.coli* BCAT (Okada et al., 2001) these residues are found to be involved in the binding of the substrate carboxyl group. The A-pocket is lined by conserved residues Arg91, Tyr29′, Phe34, Trp120, and Tyr124 as well as the non-conserved residues Leu101′ and Leu103′ (where ′ denotes-residues of another subunit).

### Ligand Binding and Enzyme Negative Cooperativity

A bound AKG molecule is clearly visible in the electron density (Figure 1) of one subunit of the GEO1900_AKG complex and has a B-factor comparable to the average for the protein. A contact analysis reveals that α-carboxylic as well as α-keto groups of the ligand are tightly bound in the active site via hydrogen bonds with the highly conserved residues Tyr89, Ala249, Thr248, Arg91 as well as Ala250 and Gly188 (via a conserved water molecule D238) (Figure S8). Although most of the residues around the γ-carboxylic group of the ligand are hydrophobic, this group is also connected to the protein via several hydrogen bonds with Arg91, Tyr29′ and Leu103′ (via the water molecule–D923).

Comparative analysis of the holo form of GEO1900 and its complex with AKG reveals that the binding of the acceptor substrate does not result in any significant conformational changes in the active site, except for the rotation of the side chains of Leu101′ and Leu103′, accompanied by a small main chain displacement (distance between corresponding Cα-atoms is about 0.6 Å). In addition the AKG binding residues 120–124 of the inter-domain loop shift toward the active site. This positions the side chains of residues Trp120 and Leu123 so that they shield the ligand from the solvent (Figures S9, S10). Interestingly, in the case of the two archaeal BCATs the residues Leu123 and Tyr124 are not part of the consensus sequence X-G-X-Y-L (where the first X is an aromatic residue) which is typical of other BCATs, rather this motif is X-G-X-L-Y in both GEO1900 and AF0933. This permutation (Tyr to Leu and *vice versa*) leads to a change in the shape of the active site cavity, making the A-pocket more extended in the direction of Leu123.

**Figure 5:**
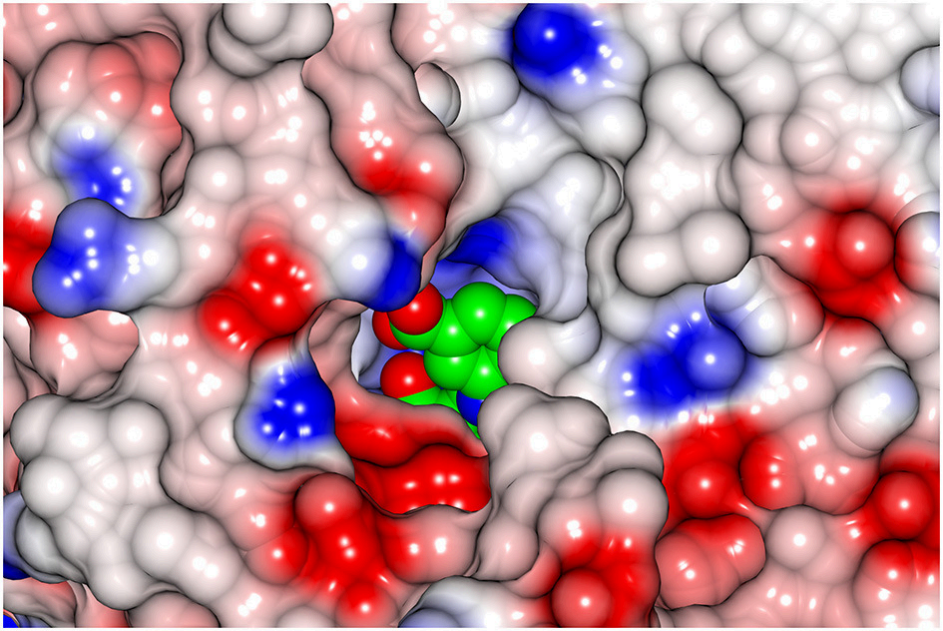
A view from outside onto the active site cavity entrance using a surface representation of the AF0933 BCAT complex with the inhibitor gabaculine (shown as spheres).

The inhibitor gabaculine makes an irreversible complex with the cofactor PLP due to the migration of the double bond of the external aldimine onto the gabaculine ring (Figure 5). However, the PLP-gabaculine adduct in the AF0933 complex structure has only partial occupancy of the gabaculine in the enzyme active site. Occupancy of the ligand in subunit F is below the level for an easy build into the model structure, therefore the internal aldimine was modeled into this subunit. The active site of the AF0933 BCAT is much more restrictive for binding of the gabaculine moiety than that of the fungal (*R*)-selective ωTAm from *Nectria haematococca* previously studied by us where it was found at 100% occupancy (Sayer et al., 2014). However, when 100:1 excess of gabaculine to PLP was added to the crystallization droplet it was expected to find full occupancy of the PLP-ligand adduct, unless AF0933 BCAT exhibits some negative cooperativity for substrate binding. Binding of the gabaculine inhibitor to one BCAT subunit appears to be sensed by neighboring subunits, with conformational adjustments preventing the binding of the inhibitor in the other subunits. Negative cooperativity has been reported earlier for other PLP enzymes (Stetefeld et al., 2006; Ruzicka and Frey, 2010) and is in agreement with the fact that the AKG binding in the GEO1900 complex structure in only found in one of the three subunits.

### Inter-domain Loop Conformation

The conformation of the loop 121–129 is significantly different between the BCAT enzymes and other class IV enzymes such as (*R*)-selective *Nectria* TAm where this loop covers the active site, creating an enclosed environment which would facilitate the activation of the amine nitrogen. In the AF0933 BCAT enzyme this loop is poorly ordered and moves away from the active site. In other BCATs including the GEO1900 BCAT this loop is closer to the active site but does not cover it as tightly as seen in the *Nectria* TAm. However, the loop appears to become more ordered in the GEO1900_AKG complex.

### Comparison of Structures of the Native Enzymes and the Complexes of GEO1900 and AF0933

From biochemical analysis it is known that AKG does not inhibit the activity of GEO1900 in the concentration range tested (Figure S11). It would appear that the crystal contacts play a role in increasing the affinity of GEO1900 toward AKG in one single subunit of the enzyme. Superposition of the subunits from the asymmetric unit gave a RMSD between the Cα atoms of about 0.2 and 0.5 Å in case of GEO1900_holo and GEO1900_AKG, respectively. In the case of the GEO1900_holo structure the conformations of the flexible loops have small differences between the different subunits. However, these are likely to be due to crystal packing effects. The structure of the complex GEO1900_AKG demonstrates that a major difference between the subunits can be assigned to a movement of the inter-domain loop which has a sufficiently different conformation in subunit A (as shown in Figure S9), where the ligand was bound, when compared to the other subunits. This loop was reported to shield the active site from the solvent upon the substrate binding (Hirotsu et al., 2005). There are two further differences between the GEO1900_AKG and GEO1900_holo structures. The first is the conformation of the loop 264–266, which differs in the A subunit when compared to subunits B and C, with a distance between the matching Cα-atoms of 1.0 Å. The second difference is in the region of residues 249–252, where the distance between corresponding Cα-atoms of the different subunits reaches 0.6 Å. As the residue displacements were only observed for the A subunit containing bound AKG, it is suggested that this could be the result of main-chain movement due to the substrate binding.

Superposition of the GEO1900_holo subunits on the GEO1900_AKG subunits reveals a further major difference in the orientation of the inter-domain loop. This loop is usually disordered in the absence of a bound substrate (Okada et al., 2001; Chen et al., 2012) with exception of the structure of the BCAT from the archaeon *T. uzoniensis* (PDB ID 5CE8; Boyko et al., 2016). In the GEO1900_holo structure the inter-domain loop is not seen in the electron density for subunits A and C, but in subunit B only two residues 120–121 are not observed (Figure S9). The other residues of the inter-domain loop have higher B factors than the average B factor of the model. It is proposed that the changes in the conformation of the inter-domain loops in GEO1900_AKG reflect the structuring of this loop during substrate binding. Thus, in the A subunit this loop adopts a conformation which shields the bound substrate from the solvent. However, in the B subunit of the complex the conformation of inter-domain loop is similar to that found in subunit B of the holo_GEO1900 structure. In the C subunit this loop is not seen in the electron density.

Although the AF0933 BCAT GABC complex was obtained by co-crystallization with gabaculine, differences in the main chain atom positions between the holoenzyme structure and the complex are minimal. No movement of the interdomain loop upon formation of the PLP-gabaculine complex was observed. The carboxyl group of the gabaculine-PLP adduct points away from both distal and proximal carboxyl group binding pockets of the BCAT. This is probably due to the orientation of this adduct being sterically hindered by the shape of the AF0933 BCAT active site cleft.

### Comparison to Structures of Other BCATs and Other CLASS IV Transaminases

**Figure 6:**
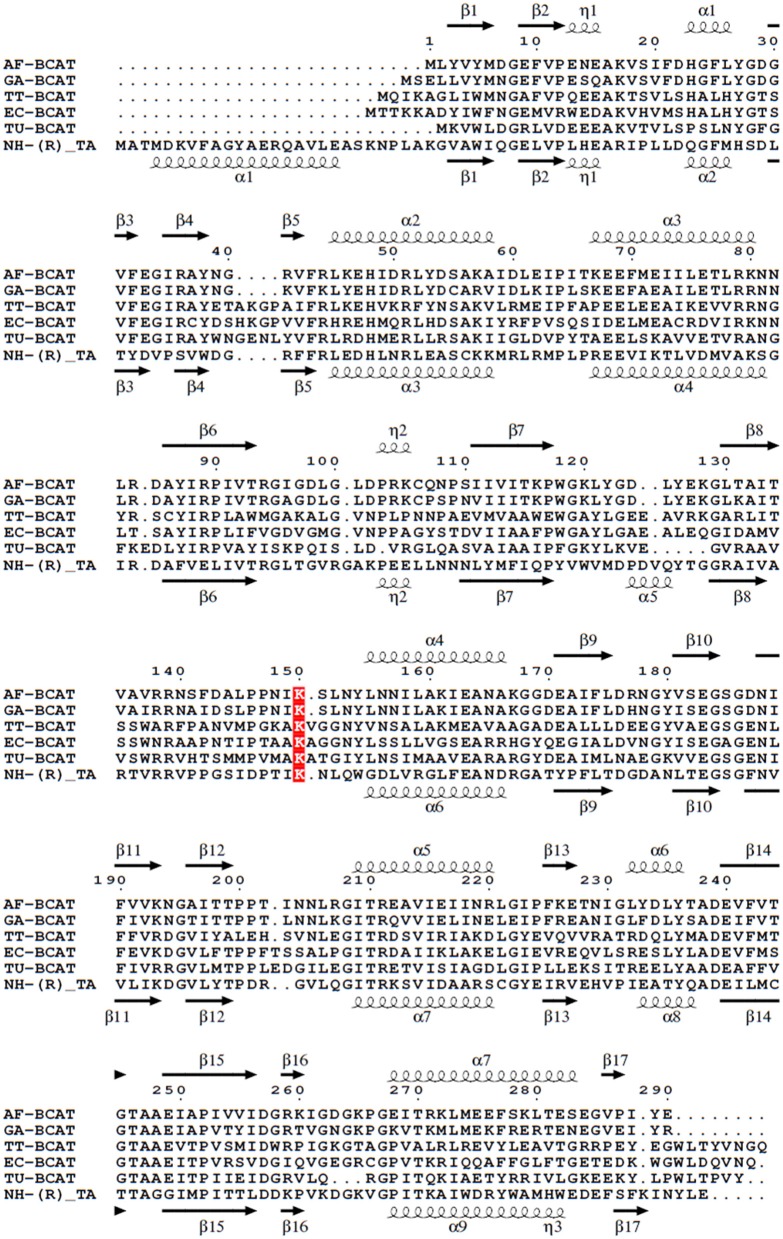
Multiple sequence alignment of different BCATs, *A. flugidus, G. acetivorans, T. thermophilus, E.coli, T. uzoniensis, N. haematococca*. Arrows indicate β-strands, and helical curves denote α-helices of the structure of AF0933 above and *Nectria* TAm below. The active site lysine is highlighted in red. The figure was prepared with ESPript3 (Robert and Gouet, 2014).

**Figure 7:**
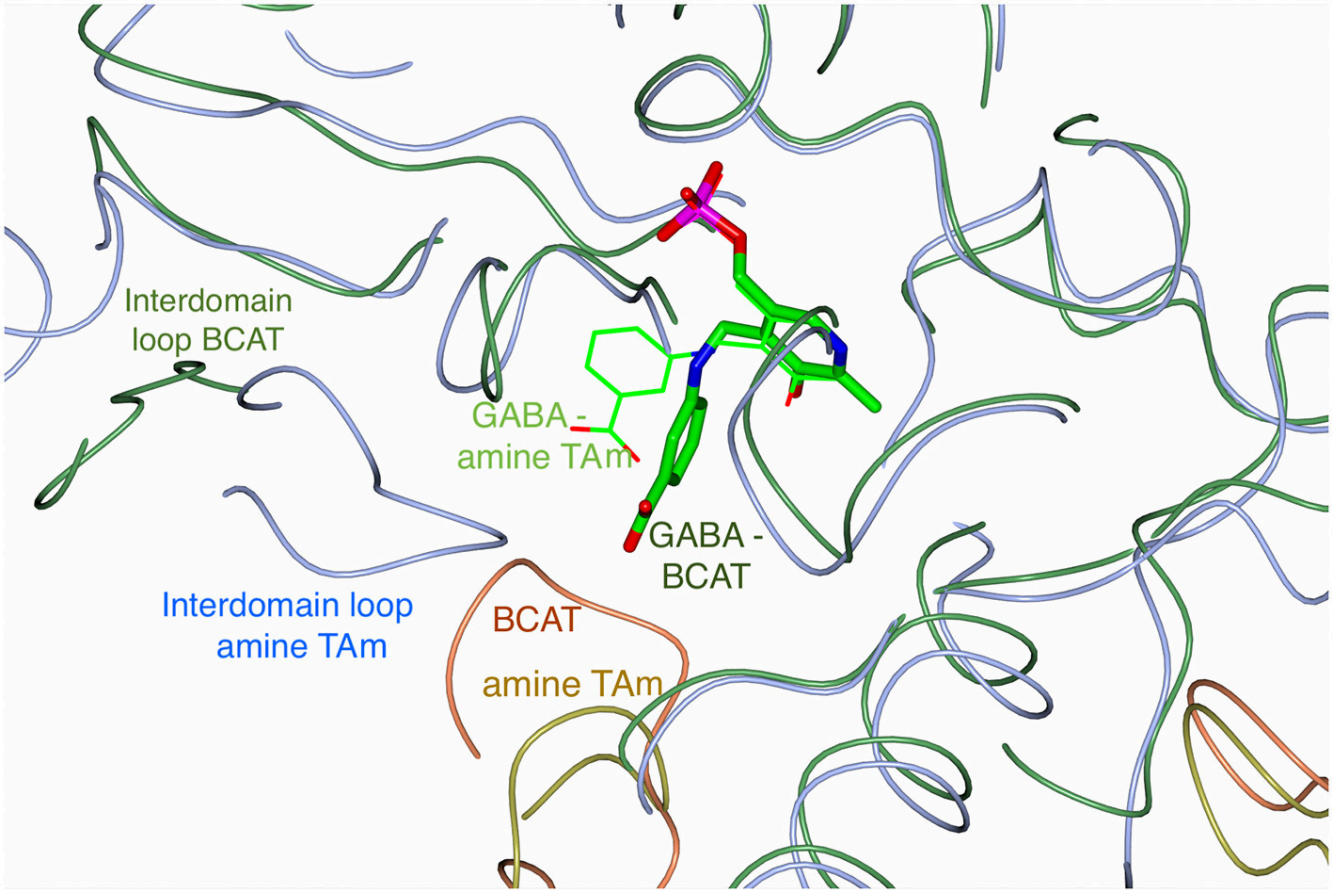
An overlay of the Cα trace of the AF0933 BCAT (green and coral coil) gabaculine complex with the *Nectria* TAm (ice blue and gold coil) gabaculine complex (Sayer et al., 2014) to illustrate the differences between the ligand and loop conformations within the active site between the two enzymes. The PLP gabaculine adduct is shown as a stick model for the AF0933 BCAT structure and as thick lines for the *Nectria* amine TAm structure. Interdomain loops and the loop connecting β5 and β6 of the N-terminal small domain of the adjacent subunit in the catalytic dimer covering the active site cavity, have different conformation between the AF0933 BCAT and the *Nectria* TAm as shown.

The BLAST search found several BCATs with known three-dimensional structure which have a moderate primary sequence similarity to GEO1900 and AF0933. The structure-based sequence alignment (Figure 6), shows the comparison of the GEO1900 and AF0933 sequences with other BCATs and another class IV TAm enzyme from the fungal species *Nectria*. This shows that the *Nectria* enzyme has an extended N-terminus and also a difference in the loop residues 99–108 (AF0933 numbering) between β5 and β6 of the N-terminal small domain. This loop contributes to the active site and is much larger in the GEO1900 and AF0933 BCATs (Figure 7).

The superposition of the GEO1900_holo subunit and the subunits of the AF0933 and other homologous enzymes using PDBeFOLD showed that the fold of the new thermostable BCATs is very similar to related enzymes. Small differences are observed within the flexible loops that connect the secondary structure elements. However, the inter-domain loop conformation is similar between GEO1900_AKG and the complexes of the *E.coli* BCAT with substrates bound (PDB codes−1I1M and 1I1L).

A comparison of the structures of GEO1900 and AF0933 has been made with other class IV TAms. The overall structures of these enzymes are similar for DATAs, BCATs and (*R*)-selective amine:pyruvate TAms. It appears that the differences are in the amino acid residues lining the active site cavity that determine substrate specificities. The BCAT catalyzed transamination between branched chain L-amino acids and the AKG acceptor produces the branched chain ketoacid and L-glutamate. The DATAs catalyze the exchange of the amino group between D-amino acids and the AKG. The (*R*)-selective amine:pyruvate TAms catalyze transfer of an amino group between a (*R*)-ω-substrate and pyruvate. Höhne et al. (2010) have identified two signature motifs which distinguish the BCAT and DATA enzymes from other (*R*)-specific amine:pyruvate TAms such as the *Nectria* TAm (Sayer et al., 2014). The conserved *Nectria* TAm residues His53, Tyr58, and Val60 belong to the first signature motif and Phe113 to the second motif. These residues contribute to the surfaces of the A and B substrate binding pockets of the active sites to enforce specific binding of the (*R*)-amine. The residues Ser62 from the first signature motif and Glu115 from the second motif, in the *Nectria* TAm prevent the BCAT activity for this enzyme. In the BCATs the equivalent residues Arg38 and Arg91 (GEO1900 numbering) are responsible for binding the α-carboxyl and the γ-carboxyl groups of the substrate.

### Structural Basis for GEO1900 and AF0933 Thermostability

Both BCATs GEO1900 and AF0933 demonstrate high thermal and solvent stability. The closest sequence homologs of these enzymes with a known 3-D structure are from another thermostable archaeal BCAT from *T. uzoniensis* (45% sequence identity to AF0933; Boyko et al., 2016), *Thermus thermophilus* (42% identity; PDB ID: 2EIY) and a mesophilic *E. coli* BCAT (40% identity; Okada et al., 2001). Thermostability of the *E. coli* BCAT does not appear to have been reported, however one can assume it is significantly lower than that of its counterparts from thermophilic organisms, although in some cases such presumption may be erroneous. For instance, the *E. coli* tryptophanase has high thermostability and was purified using a heat treatment step (Dementieva et al., 1994).

Features that are known to confer increased thermostability of proteins include an increase in ionic interactions or salt bridges which offer more thermostability when found in clusters, increased hydrophobicity and the shortening of surface loops (Littlechild et al., 2013). The AF0933 TAm subunit forms up to 27 ion pairs, with five 3-amino acid salt bridge clusters formed. There are no ion pairs between the subunits in the catalytic dimer, however three ion pairs per subunit are formed with neighboring subunits within the hexamer. Residues with multiple side chain conformations in many cases form ion pairs with different residues for either conformation. The *E.coli* BCAT has 15 ion pairs altogether, including those involved in four 3-amino acid clusters. None of these are with neighboring subunits. Most of salt bridges and other structural features observed in the AF0933 are conserved in GEO1900, therefore comparison here is limited to the former protein.

The AF0933 BCAT has much shorter loops connecting secondary structure elements in comparison with the *E. coli* BCAT. Particularly shortened are the loops connecting strands β3 to β4 and β5 to β6 of the N-terminal domain and β5 toβ6 of the large domain. The C-terminal inter-domain loop, which is only 2 residues long in the AF0933 BCAT, is 9 residues long in the *E. coli* enzyme. Shorter loops also mean that there is a higher percentage of residues belonging to the secondary structure, 55.9% in AF0933 BCAT compared to 49.2% in the *E. coli* BCAT. The inter-subunit interface in the catalytic dimer is mainly hydrophobic both in the AF0933 and in the *E. coli* BCAT, therefore it appears that hydrophobic interactions contribute equally to the stability of both proteins.

Thus, an increased number of ion pairs per subunit alongside shorter surface loops are the main contributing factors to the increased thermostability of AF0933 when compared to the *E.coli* BCAT.

### Application to Biocatalysis

**Figure 8:**
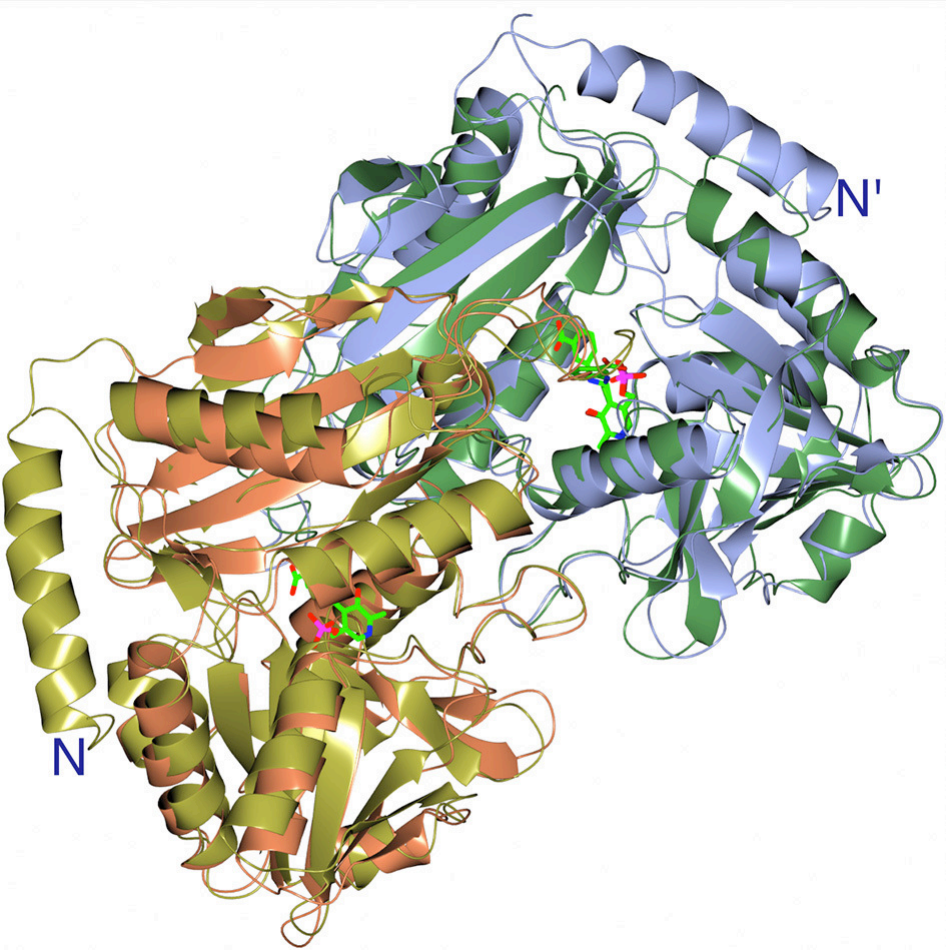
A cartoon representation of the superimposition of the catalytic dimers of the AF0933 gabaculine complex with the *Nectria* TAm gabaculine complex, illustrating the additional α-helix at the N-terminal region of *Nectria* TAm as shown in Figure 6. The PLP gabaculine adduct is shown in ball and stick mode.

Both GEO1900 and AF0933 make a good thermostable and solvent tolerant scaffold which can be optimized for production of natural and synthetic chiral amines of interest to the pharmaceutical industries. The new BCAT enzymes have a chiral preference for the (*R*)-isomer of MBA but with low activity. However, they offer the possibility to be further optimized for this and other substrates of interest using the knowledge of the active site cavity obtained from the current structural studies. The active site cavities of GEO1900 and AF0933 can be extended to better accommodate MBA and other bulky substrates. The comparison of GEO1900 and AF0933 with a (*R*)-specific TAm from a *Nectria* sp. shows that this fungal enzyme has an additional long N-terminal α-helix that increases the depth of the active site of this enzyme. The presence of this N-terminal helix shown in Figure 8, a much tighter closure over the active site of the interdomain loop, and a different conformation of the loop between β5 and β6 of the N-terminal small domain, creates a difference in the substrate environment of the *Nectria* TAm in comparison with that in the GEO1900 and AF0933 BCATs. This would potentially alter the local dielectric constant within the fungal enzyme active site thereby facilitating the observed amination reactions of ω-keto acids and different aldehydes and ketones by this TAm (Sayer et al., 2014).

## Conclusions

The approach developed in the THERMOGENE project was to search for these new archaeal TAm enzymes within the untapped resource of “natures” biodiversity in order to find enzymes that have evolved to be active under the extreme growth conditions of their host organisms. This approach has been successful and has resulted in the discovery of new thermostable BCATs displaying new features which would have been missed in a directed evolution approach using already identified mesophilic enzymes. The thermophilic archaea *G. acetivorans* and *A. fulgidus* were isolated from different hydrothermal sources. The *G. acetivorans* has been isolated from samples of deep-sea hydrothermal vents of the Ashadze field, the deepest of the known World Ocean hydrothermal fields (Querellou et al., 2009) whereas the *A. fulgidus* was isolated in shallow hyperthermal sea water vents in Volcano Island in Sicily (Stetter, 1988) yet they have related BCAT enzymes with 79% sequence identity and similar properties. The GEO1900 and AF0933 enzymes have been characterized both biochemically and structurally. They both demonstrate significant thermostability and organic solvent stability and are promising candidates for industrial applications. Both the AF0933 and the GEO1900 are very thermostable as determined by the stability trials conducted. The AF0933 is a hexamer in solution as measured by gel filtration studies whereas the GEO1900 is mainly found as a dimer. The crystallographic studies have provided structures of both enzymes in the internal aldimine form, to 1.9 Å resolution for the *Geoglobus* enzyme and 2.0 Å for the *Archaeoglobus* enzyme. The structures of the enzymes with substrate and inhibitor have revealed the conformational changes occurring in the enzymes during catalytic turnover. A comparison of the *Archaeoglobus* inhibitor complex structure with the related (*R*)-selective fungal *Nectria* enzyme structure has produced further insights into differences in substrate specificity between the two enzymes. The new robust (*R*) selective BCAT enzymes will add to the enzyme “tool box” available for industrial biocatalysis and will have applications for production of new important chiral intermediates for the pharmaceutical industries using a sustainable route.

## Materials and Methods

All reagents were purchased from Sigma–Aldrich, Buchs, Switzerland unless otherwise stated. The chromatography columns were obtained from GE Healthcare (Little Chalfont, UK).

### Expression and Purification

One gene encoding a class IV transaminase was identified in the complete genome sequence of *G. acetivorans* (Mardanov et al., 2011) by the BLAST search (Altschul et al., 1990). The nucleotide sequence of this gene was synthesized for optimal expression in *E. coli*. The GEO1900 was cloned into the *Bam*HI and *Hind*III sites of the expression vector pQE30 (Qiagen, Manchester, UK). The encoded recombinant protein contained an N-terminal MRGSHHHHHHGS tag. The *E. coli* DLT1270 cells harboring the expression vector pQE30_GEO1900 were grown in LB medium at 37°C, containing 100 μg ml^−1^ ampicillin, to an OD_600_ of 0.5. Protein expression was induced by addition of 1 mM Isopropyl-β-d-thiogalactopyranoside (IPTG) for 19 h at 37°C. The cells were harvested by centrifugation. The cell paste was resuspended in 50 mM Tris-HCl buffer, pH 7.5, containing 500 mM NaCl, 2 M urea, 20 mM imidazole, 2 mM β-mercaptoethanol, 0.1% (v/v) Triton X-100, and 1 mM phenymethylsulfonyl fluoride (PMSF). After the cells were disrupted by sonication the crude cell extract was centrifuged at 10,000 g for 25 min. The filtered supernatant was applied to a Hi-Trap chelating HP column (2 ml) equilibrated in 50 mM Tris-HCl, pH 7.5, containing 500 mM NaCl, 20 mM imidazole, and 0.1% (v/v) Triton X-100. The (His)_6_-tagged recombinant GEO1900 was eluted with a linear gradient from 20 to 500 mM imidazole in the same buffer but without Triton X-100. The active fractions were pooled and incubated with 1 mM PLP for 1 h at 40°C, concentrated using a 30 kDa cut-off centrifugal filter device (Millipore), and applied to a Superdex 200 column (GE Healthcare) equilibrated in 30 mM Tris-HCl, pH 8.0, supplemented with 300 mM NaCl. The fractions containing the holo GEO1900 protein were stored at −20°C with the addition of 50% glycerol. The protein purity was analyzed by SDS-PAGE (12%). The protein concentration was determined spectrophotometrically using the calculated extinction coefficient of 24,955 M^−1^ cm^−1^.

Another class IV transaminase gene was identified by searching known thermophilic archaeal genomes using the BCAT from *N. haematococca* as a query sequence. This identified a 290 amino acid protein in *A. fulgidus* (Klenk et al., 1997). The nucleotide sequence of this gene was synthesized for optimal codon usage for expression in *E. coli*. The expression of recombinant AF0933 was carried out using the plasmid pLATE51-AF0933 (Thermo Fisher Scientific, Ashford, UK) which was transformed into *E. coli* Rosetta (DE3)-pLysS (Novagene). Cells were grown in 1.6 l LB medium at 37°C to an OD_600_ of 0.4–0.6 and expression was started by the addition of 1 mM IPTG. After 4.5 h of further growth, cells were harvested by centrifugation (max 9,800 × g at 18°C). The *E. coli* pellet was suspended in 50 mM Tris-HCl, pH 8.2, containing 300 mM NaCl and 10 mM imidazole (buffer A). Cell disruption was performed by passing through a French pressure cell (16,000 psi) followed by centrifugation (max 48,000 × g at 4°C). The supernatant was incubated for 20 min at 70°C to precipitate *E.coli* proteins and centrifuged (max 48,000 × g at 4°C). The resultant supernatant was applied to a 1 ml nickel-nitrilotriacetic acid (Ni-NTA) column (Qiagen, Manchester, UK) equilibrated in buffer A using the ÄKTA FPLC system. After a washing step, protein was eluted by stepwise increase of the imidazole concentration up to 500 mM. The fraction containing the highest enzyme activity was applied to a Superdex 200 HiLoad size exclusion column (1.6 × 60 cm) equilibrated with 50 mM Tris-HCl, pH 7.5, containing 500 mM NaCl and 0.05 mM PLP (GE Healthcare, Little Chalfont, UK). The protein purity was analyzed by SDS-PAGE (12%). Protein concentration was determined by using the Bradford method (Bradford, 1976) or spectrophotometrically for the purified protein.

### Enzyme Activity Assays

The overall transamination reaction of GEO1900 was assayed by a discontinuous method by measuring the rate of formation of L-glutamic acid from AKG in the presence of BCAAs in the direct reaction or the formation of AKG from L-glutamate acid in the presence of keto acids in the reverse reaction. In the direct reaction assay GEO1900 (5–30 μg) was pre-incubated in 1 ml of the reaction mixture, containing 50 mM phosphate buffer, pH 8.0, 100 mM NaCl, 60 μM PLP, and 5 mM of the amino acid at 65°C for 3 min. The reaction was initiated by the addition of 5 mM AKG. Samples (100–300 μl) were taken at different time points and frozen to stop the reaction. Then the L-glutamic acid concentration was evaluated in the samples after dilution to 1 ml by spectrophotometry employing the glutamate dehydrogenase (GluDH) assay at 25°C in 50 mM Tris-HCl buffer, pH 9.0, supplemented with 1 mM NAD and 0.5 U GluDH. In the reverse reaction assay the enzyme (5–30 μg) was preincubated in the reaction mixture (1 ml) containing 50 mM phosphate buffer, pH 8.0, 100 mM NaCl, 60 μM PLP, and 5 mM L-glutamic acid at 65°C for 3 min. The reaction was initiated by the addition of 5 mM ketoacid. Samples (100–300 μl) were taken at several time points and frozen to stop the reaction. The α-ketoglutarate concentration was evaluated in the samples after dilution to 1 ml by spectrophotometry employing the GluDH assay at 25°C in 20 mM sodium phosphate buffer, pH 7.3, supplemented with 0.09 mM NADH, 30 mM ammonium chloride and 0.5 U GluDH. The specific activity of GEO1900 was calculated from the initial linear region of the progress curve of the L-glutamic acid/ α-ketoglutarate formation.

Steady-state kinetic parameters for pyruvate and AKG were determined from the substrate saturation curves for the overall reaction between the keto substrate and 5 mM L-Leu as a co-substrate. The saturation curves were analyzed using the Michaelis-Menten model. The GEO1900 activity toward (*R*) and (*S*)-MBA was measured spectrophotometrically at 65°C by the acetophenone assay (Schätzle et al., 2011; Boyko et al., 2016).

The AF0933 BCAT activity was measured as 3-methyl-2-oxobutyrate dependent formation of AKG from L-glutamate. One unit of enzyme activity corresponded to the conversion of 1 μmol of substrate consumed or product formed per min. The standard assay mixture contained 0.1 M Tris-HCl, pH 8, 0.1 mM PLP, 5 mM 3-methyl-2-oxobutyrate, 10 mM L-glutamate and protein. Samples were incubated at 70°C up to 40 min, the reaction was stopped on ice and the amount of AKG formed was quantified by measuring NADH consumption in the reductive amination of AKG to L-glutamate at 340 nm. The quantification mixture (0.3 ml) was incubated at 20°C for 10 min and contained 0.1 M Tris-HCl, pH 7.5, 0.3 mM NADH, 40 mM NH_4_Cl, 5–100 μl of the samples and 0.32 U of L-glutamate dehydrogenase. Protein was determined by the Bradford method.

Kinetic constants for 3-methyl-2-oxobutyrate, 4-methyl-2-oxopentanoate and 3-methyl-2-oxopentanoate were determined with substrate concentrations up to 15 mM, 10 mM L-glutamate and 0.7–4 μg protein using standard conditions. Kinetic constants for L-glutamate were determined with substrate concentrations up to 80 mM, 5 mM 3-methyl-2-oxobutanoic acid and 2.7–9.1 μg protein using standard conditions. The substrate specificity was tested with 1 or 10 mM amino acceptors, 2.5 μg protein using standard conditions.

The reverse reaction was measured as L-valine dependent decrease of AKG. The assay mixture contained 0.1 M Tris-HCl, pH 8.0, 0.1 mM PLP, 10 mM L-valine, 10 mM AKG and 5.3 μg protein. Standard conditions were used for further incubation and quantification.

AF0933 activity was also measured as 3-methyl-2-oxobutyrate dependent formation of pyruvate from L-alanine. The assay mixture contained 0.1 M Tris-HCl, pH 8.0, 0.1 mM PLP, 5 mM 3-methyl-2-oxobutyrate, up to 20 mM L-alanine and 2.7 μg protein. Samples were incubated at 70°C up to 20 min, the reaction was stopped on ice and the amount of pyruvate formed was quantified by measuring the reduction of pyruvate to lactate at 340 nm. The quantification mixture (0.3 ml) was incubated at 20°C for 10 min and contained 0.1 M Tris-HCl, pH 7.5, 0.3 mM NADH, 15–100 μl of the samples and 1.8 U of L-lactate dehydrogenase (Roche, Burgess Hill, UK).

The AF0933 activity with (*R*) and (*S*)-MBA was determined by the acetophenone assay. The assay mixture of 1 ml contained 0.1 M Tris-HCl, pH 8.0, 0.1 mM PLP, 2 mM 3-methyl-2-oxobutyrate, 2-oxobutyrate or pyruvate as amino acceptor, 2 mM (*R*)-MBA or (*S*)-MBA as amino donor and 16 μg protein. Samples were incubated at 65°C up to 180 min, the reaction was stopped on ice and the absorbance was measured at 245 nm. The molar extinction coefficient of 11.6 mM^−1^ cm^−1^ was used for acetophenone.

### Effects of pH, Temperature, and Solvents on the Transamination Reaction

The pH optimum of the reaction for GEO1900 with 5 mM L-Leu, and 5 mM AKG was determined at 65°C using 50 mM sodium phosphate, pH 6.0–8.0, 50 mM BICINE containing 100 mM NaCl.

The temperature optimum of the transamination reaction between 5 mM L-Leu, and 5 mM AKG was determined in the temperature range from 40 to 95°C in 50 mM phosphate buffer, pH 7.9, supplemented with 100 mM NaCl. The thermostability of GEO1900 was determined by incubating a 1.0 mg/mL enzyme samples in 100 mM phosphate buffer, pH 8.0 at different temperatures between 30 and 70°C for 2 h. After the thermal treatment, the remaining enzyme activity was determined at 65°C as described above.

The pH optimum of AF0933 was determined with 3.6 μg protein, standard conditions and the following buffers each at a concentration of 0.1 M: MES (pH 5.5, 6.0, 6.5), Bis-Tris (pH 6.5, 7.0), HEPES (pH 7.0, 7.5, 8.0), Tris-HCl (pH 7.5, 8.0, 8.5), Bicine (pH 8.5, 9.0) and Piperazine (pH 9.0, 9.5, 10.0).

The temperature optimum of the reaction of AF0933 was determined between 50°C and 95°C in 0.1 M HEPES, pH 7.0, containing 0.1 mM PLP, 5 mM 3-methyl-2-oxobutyrate, 10 mM L-glutamate and 2.7–5.4 μg protein.

The thermostability of AF0933 was measured between 55 and 95°C, therefore protein was incubated in 3 volumes of buffer (0.1M HEPES, pH 7.0 adjusted to the respective temperature) up to 120 min. Samples (2.8 μg protein) were chilled on ice and remaining BCAT activity was determined in 0.1 M HEPES, pH 7.0 using standard conditions.

The influence of solvents (ethanol, DMSO, methanol and acetonitrile) on activity was analyzed between 10 and 50% solvent. The respective solvents were incubated with 2.2 μg protein (for AF0933) and 50 μg protein (for GEO1900) for 1 h at 20°C and 55°C then remaining activity was measured with standard conditions.

### Protein Crystallization

For crystallization fractions of GEO1900 after the size-exclusion chromatography step were concentrated up to 10 mg/ml using an Amicon Ultra 30 kDa MWCO (Merck Millipore, Darmstadt, Germany) centrifugal filter units and stored at −70°C in 30 mM Tris-HCl buffer, pH 8.0, supplemented with 300 mM NaCl and 100 μM PLP. High-throughput crystallization screening was setup using a robotic system (Rigaku Automation, USA) at the Resource Centers division of the NBICS Centre of the National Research Centre “Kurchatov Institute” (Boyko et al., 2013). The sitting-drop vapor-diffusion method was applied at 20°C in 96-well CrystalMation plates (Rigaku Automation, USA). The first round of the screen resulted in three in the following conditions: (1) 0.2 M TMAO, 0.1 M Tris-HCl, pH 8.5, 20% PEG 2000MME; (2) 30 mM citric acid, pH 3.0; 70 mM Bis-tris propane, 20% PEG 3350; (3) 0.2 M KCl; 0.1M ammonium sulfate, pH 7.5; 20% 2-propanol, pH 7.5. Crystals grown under conditions (1) and (2) had rod-like shape with bad surface morphology and tended to form clusters. Under condition (3) numerous cube—like crystals were grown with the dimensions of 20 × 20 × 20 μm. The further optimization was made using the hanging-drop vapor-diffusion technique in 24-well VDX plates (Hampton Research, Aliso Viejo, California, USA) at 20°C by varying ammonium sulfate concentration, pH of the reservoir solution and addition of organic compounds. Crystals suitable for X-ray diffraction appeared in 3 months in the 3 μl drop with protein to precipitant ratio of 1:1 equilibrated over 500 μl of reservoir solution. The crystals used for data collection grew in (1) 0.1 M Tris-HCl pH 8.0, 12% glycerol, 1.5 M ammonium sulfate, and (2) 0.1 M HEPES, pH 7.5; 0.7M lithium sulfate. Crystals of GEO1900 grown under condition (1) (GEO1900_holo) were briefly soaked in mother liquor containing 30% glycerol as a cryoprotectant. To obtain the complex of GEO1900 with AKG crystals from condition (2) were soaked for 20 min in the mother liquor supplemented with 50 mM AKG (GEO1900_AKG) then transferred into a cryoprotectant containing the same solution with addition of 30% ethylene glycol. Crystals were flash-cooled to 100 K in liquid nitrogen immediately prior to the X-ray experiment.

Prior to crystallization the AF0933 enzyme was concentrated to ~10 mg ml^−1^ using a 10 kDa Vivaspin membrane (Vivaproducts, Littleton, Massachusetts, USA). Microbatch crystallization trials were set up using an Oryx 6 crystallization robot (Douglas Instruments, Hungerford, England) using the JCSG+ screen (Molecular Dimensions, Newmarket, England) (Newman et al., 2005). The droplet consisted of a 50:50 ratio of protein solution to screen solution and was covered with Al's oil (50:50 mixture of silicone oil and paraffin). The crystallization plates were stored at 18°C and were regularly checked for appearance of crystals under a light microscope.

Crystals of the AF0933 holoenzyme were grown using several different PEG and methylpentanediol (MPD) conditions. The best crystals grew in 100 mM cacodylate buffer pH 6.5, 200 mM magnesium chloride and 50% v/v PEG200. To obtain the inhibitor bound complex AF0933 was co-crystallized with 5 mM gabaculine under the same conditions.

### Data Collection and Processing

The GEO1900 data were collected at the BL41XU beamline of the Spring8 synchrotron (Harima, Japan) using a Pilatus detector. The data were indexed, integrated, and scaled using XDS (Kabsch, 2010) for the holo enzyme and using Mosflm (Battye et al., 2011) for the complex with AKG. The data showed both structures were not twinned, based on the L-test (Padilla and Yeates, 2003). The program Pointless (Evans, 2006) suggested the P3_1_21 space group or its enantiomorph P3_2_21.

Data for AF0933 were collected on beamline I04-1 at the Diamond Synchrotron light source (Oxford, UK) under cryo conditions (100 K in a stream of gaseous nitrogen). Data were processed using XDS (Kabsch, 2010) and AIMLESS (Evans and Murshudov, 2013) in the Xia2 pipeline (Winter et al., 2013). Further data and model manipulation was carried out using the CCP4 suite of programs (Winn et al., 2011). The data collection and processing statistics for both GEO1900 and AF0933 are summarized in Table 3.

### Structure Solution and Refinement

The structure of GEO1900_AKG was solved at 2.2 Å resolution by the molecular replacement (MR) method using the BALBES MR pipeline (Long et al., 2007), the MR search was carried out in both enantiomorph space groups. The solution was found in P3_2_21 with the atomic model of BCAT from *T. thermophilus* (PDB ID: 2EIY), which shares 42% of amino acid sequence identity with GEO1900, and was refined to an *R*-free of 37.7% without any model rebuilding, the BALBES estimation of probability of correct solution was 99%. The GEO1900_holo structure was solved to 2.2 Å by MR with the atomic coordinates of GEO1900_AKG.

The AF0933 holo-structure was phased by MR implemented in MOLREP (Vagin and Teplyakov, 2010) using a sequence modified model (Lebedev et al., 2008) of a dimer of *N. haematococca* TAm [31% sequence identity; PDB ID 4CMD; (Sayer et al., 2014)] as a model. The gabaculine inhibitor bound complex of AF0933 crystallized in the same space group P2_1_2_1_2_1_.

Refinement of all structures was carried out with REFMAC5 (Murshudov et al., 2011). The manual rebuilding of the models into electron density maps was carried out with the COOT interactive graphics program (Emsley et al., 2010). The final refinement cycles of the GEO1900_holo structure were conducted with inclusion of hydrogen atoms in riding positions. The 6-fold NCS averaging by DM (Cowtan, 2010) was used for phase improvement of AF0933 structures. The resulting phases from density modification were used for phase refinement in REFMAC5 (Pannu et al., 1998). BUSTER refinement maps (Bricogne et al., 2016) were used for building better positioning of poorly defined loops and of the inhibitor with partial occupancy in the AF0933 active site.

### Structure Analysis and Validation

PROCHECK was used for assessment of the quality of the models (Laskowski et al., 1993). Images were created with CCP4mg (McNicholas et al., 2011) and PyMOL molecular graphics system (Schrödinger, LLC). A visual inspection of the models was performed using the PyMOL and COOT software. The BLAST service (Altschul et al., 1990) was used for the amino acid alignment. The structure superposition was carried out using the PDBeFOLD program (Krissinel and Henrick, 2004). The contacts were analyzed using WHATIF (Vriend, 1990) and PISA (Krissinel and Henrick, 2007) software. The ligand coordination was visualized by LigPlot (Laskowski and Swindells, 2011).

The atomic coordinates and structure factors for both GEO1900 and AF0933 have been deposited in the Protein Data Bank. The PDB codes for GEO1900 holo enzyme as 5CM0 and GEO1900 complexed with AKG 5E25. The PDB codes for AF0933 holo enzyme 5MQZ and AF0933 complexed with gabaculine 5MR0.

## Author Contributions

EB, NR, AM, PJ, and CS performed the discovery, cloning, and expression of the novel enzymes, while EB, PJ, CS, AN, and KB performed the protein purification, crystallization, and structure determination studies with MI. KB carrying out data analysis and structural refinement. AN, TS, MS, and J-MS performed the biochemical characterization of functional properties and the substrate specificity studies with PS. JL, VP, PS, MI, and EB coordinated the work and wrote the manuscript with input from all authors.

### Conflict of Interest Statement

The authors declare that the research was conducted in the absence of any commercial or financial relationships that could be construed as a potential conflict of interest.
