# Corrigendum: Thermostable Branched-Chain Amino Acid Transaminases From the Archaea *Geoglobus acetivorans* and *Archaeoglobus fulgidus*: Biochemical and Structural Characterization

**DOI:** 10.3389/fbioe.2019.00079

**Published:** 2019-04-16

**Authors:** Michail N. Isupov, Konstantin M. Boyko, Jan-Moritz Sutter, Paul James, Christopher Sayer, Marcel Schmidt, Peter Schönheit, Alena Yu. Nikolaeva, Tatiana N. Stekhanova, Andrey V. Mardanov, Nikolai V. Ravin, Ekaterina Yu. Bezsudnova, Vladimir O. Popov, Jennifer A. Littlechild

**Affiliations:** ^1^Henry Wellcome Building for Biocatalysis, Biosciences, University of Exeter, Exeter, United Kingdom; ^2^Research Center of Biotechnology of the Russian Academy of Sciences, Moscow, Russia; ^3^Institut für Allgemeine Mikrobiologie, Christian-Albrechts-Universität Kiel, Kiel, Germany

**Keywords:** thermophilic archaea, branched-chain aminotransferases, substrate specificity, X-ray structural analysis, biocatalysis

An author name was incorrectly spelled as “Peter Schoenheit.” The correct spelling is “Peter Schönheit.”

The authors apologize for this error and state that this does not change the scientific conclusions of the article in any way. The original article has been updated.

