# Supplementary material for: Thermostable Branched-Chain Amino Acid Transaminases From the Archaea Geoglobus acetivorans and Archaeoglobus fulgidus: Biochemical and Structural Characterization
Source: Front Bioeng Biotechnol. 2019 Jan 24;7:7. doi: 10.3389/fbioe.2019.00007 (PMC6353796; doi:10.3389/fbioe.2019.00007)

SUPPLEMENTARY

Table S1

Substrate specificity of GEO1900 towards amino donors

Amino donors	U-mg^{-1}	% activity
L-leucine	13.60	100
L-isoleucine	14.20	104.4
L-tert-leucine	2.05	15.1
L-valine	17.35	127.6
L-norleucine	9.2	67.6
L-norvaline	1.6	11.8
L-phenylalanine	2.3	16.9
L-ornithine	nd	-
2-aminobutyrate	1.8	13.2
L-histidine	0.67	4.9
DL-lysine	nd	-
L-alanine	nd	-
D-alanine	nd	-
β-alanine	nd	-
L-cysteine	0.44	3.2
L-tyrosine	nd	-

The specificity for amino donors was evaluated at 65 °C in 50 mM phosphate buffer, pH 8.0, supplemented with 100 mM NaCl and 60 µM PLP using 5 mM amino acid and 5 mM α-ketoglutarate. 100% relative activity refers to 13.64 ± 0.79 U mg^{-1} of activity under the tested conditions

Figure S1. The gel filtration elution profile for the BCAT from *Geoglobus acetivorans* (GEO1900). Column size and specifications- A calibrated Superdex 200 column (GE Healthcare) bed volume 24 ml equilibrated in 50 mM Tris-HCl, pH 8.0, 100 mM NaCl, 0.02 mM PLP. The major peak corresponds to a dimer for the native GEO1900 enzyme.

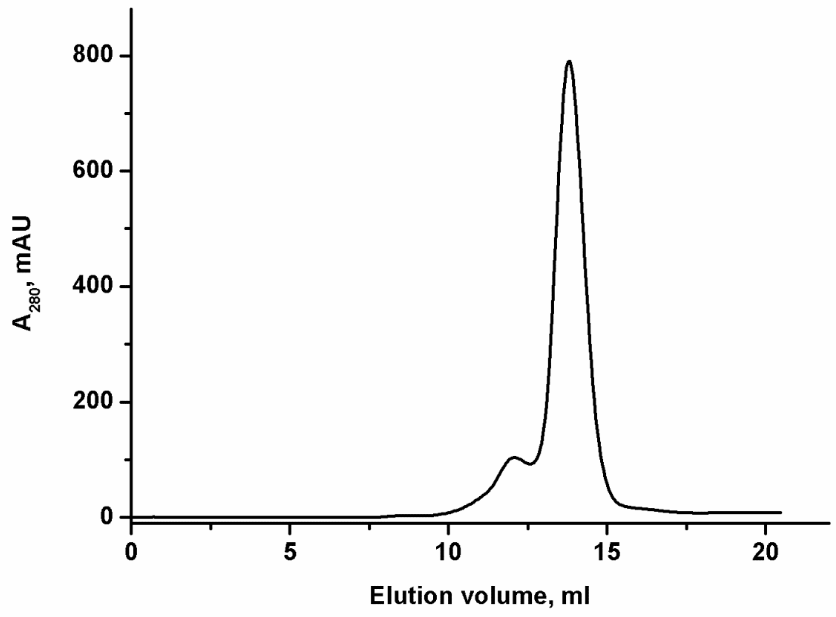

Figure S2. The gel filtration elution profile for the BCAT from *Archaeoglobus fulgidus* (AF0933). Column size and specifications- A calibrated Superdex 200 HiLoad size exclusion column (GE Healthcare) bed volume 131ml equilibrated with 50 mM Tris-HCl, pH 7.5, containing 500 mM NaCl and 0.05 mM PLP**.** The major peak corresponds to a hexamer for the native AF0933 enzyme.

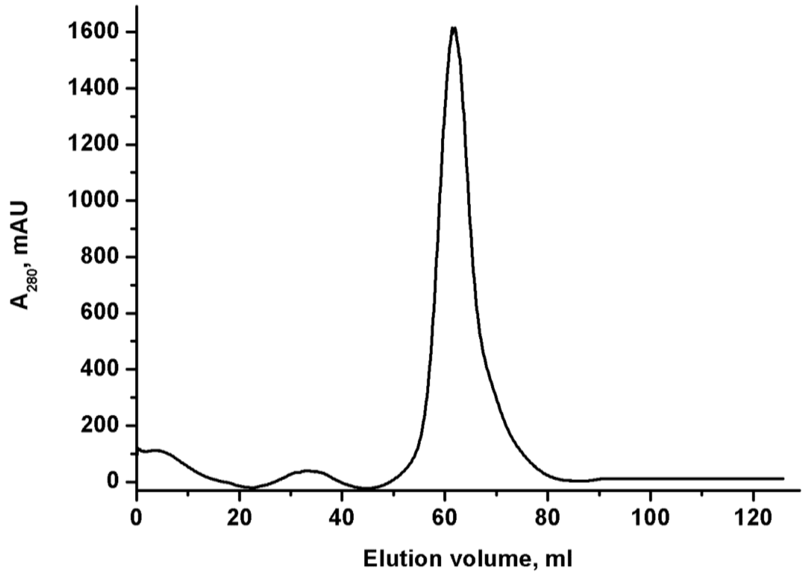

Figure S3. The effect of temperature on the activity of the GEO1900 BCAT in the reaction with L-Leu and α-ketoglutarate in 50 mM phosphate buffer, pH 7.9, 100 mM NaCl.

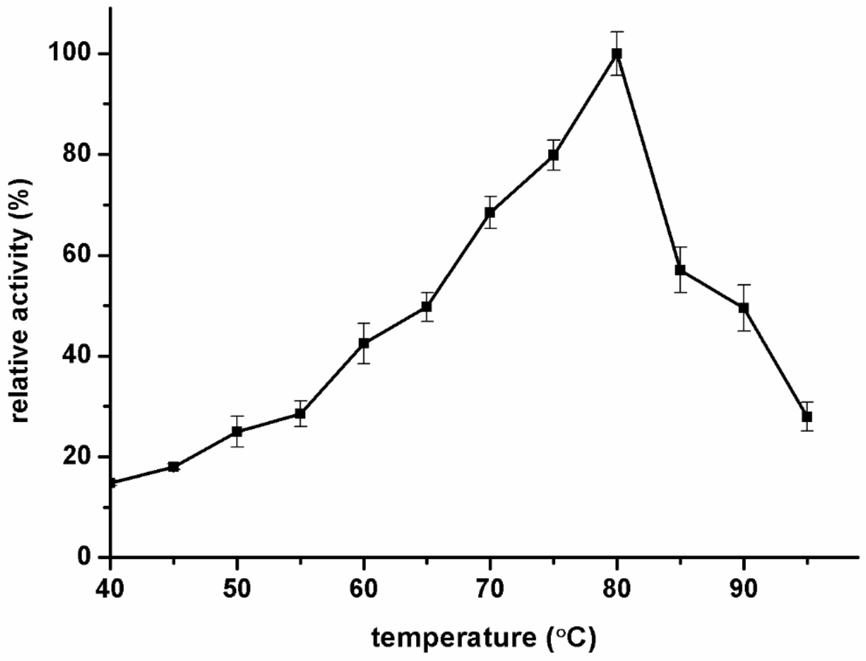

Figure S4. The thermostability of the GEO1900 BCAT after incubation up to 120 min at temperatures of 30°C (o), 50ºC (▲) and 70ºC cooling on ice and assaying under standard conditions.

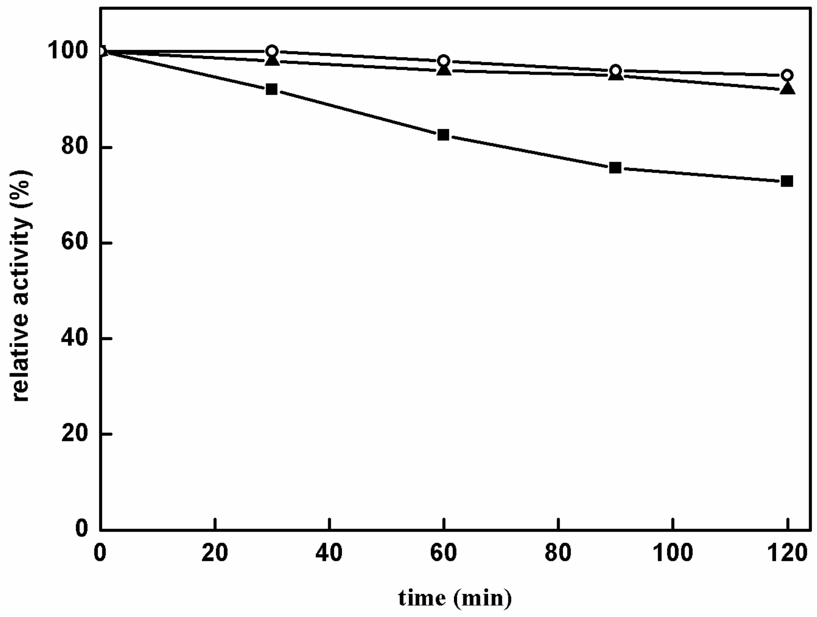

Figure S5. Effect of temperature on the specific activity of the AF0933 BCAT

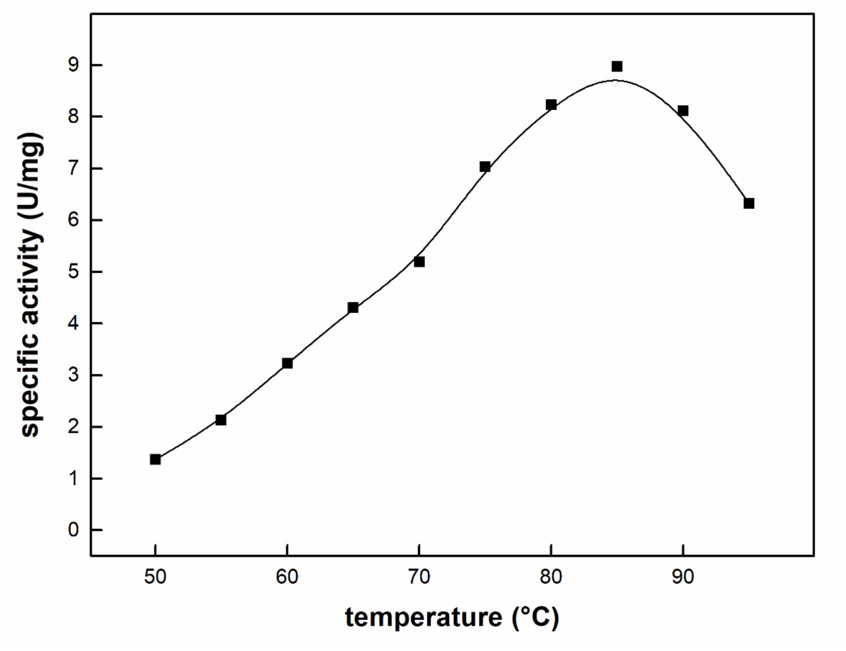

Figure S6. The thermostability of the AF0933 BCAT after incubation for up to 120 min at 55°C (■), 65°C (●), 75°C (▲), 85°C (▼) and 95°C (♦) cooling on ice and assaying under standard conditions.

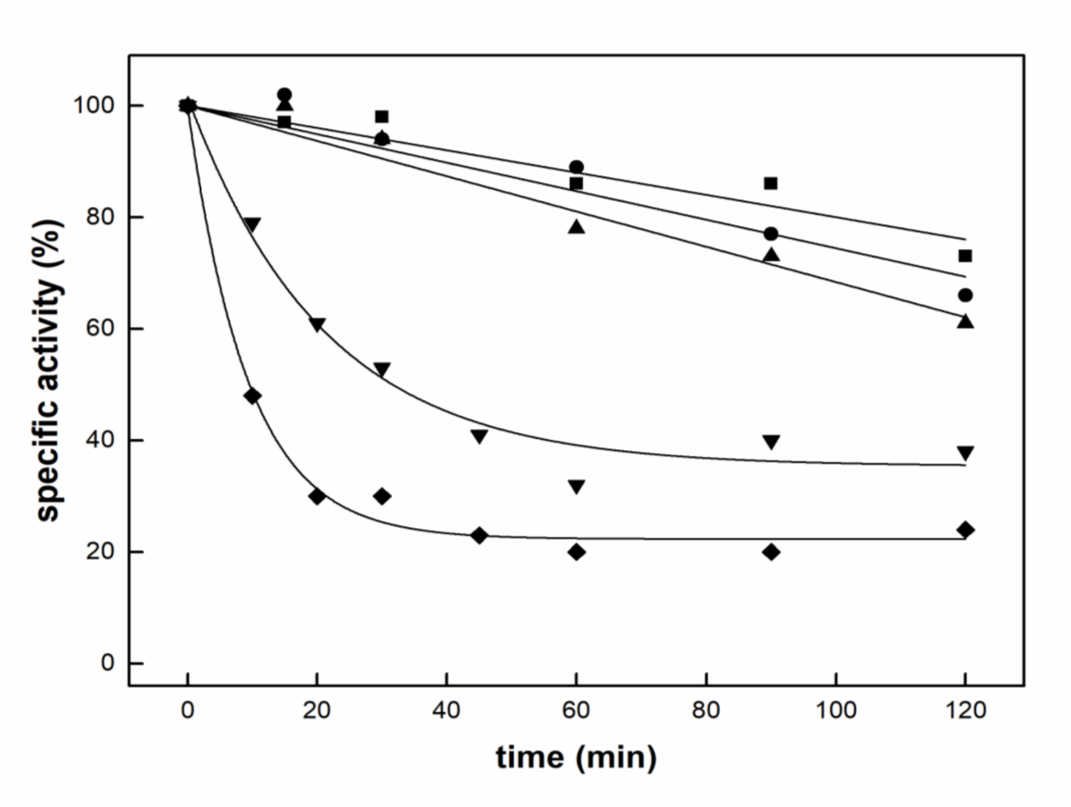

Figure S7. Rate dependence of AF0933 on the concentrations of L-glutamate (a), 3-methyl-2-oxopentanoate (b), 3-methyl-2-oxobutyrate (c) and 4-methyl-2-oxopentanoate (d). The insets show double reciprocal plots of the rates vs the corresponding substrate concentrations.

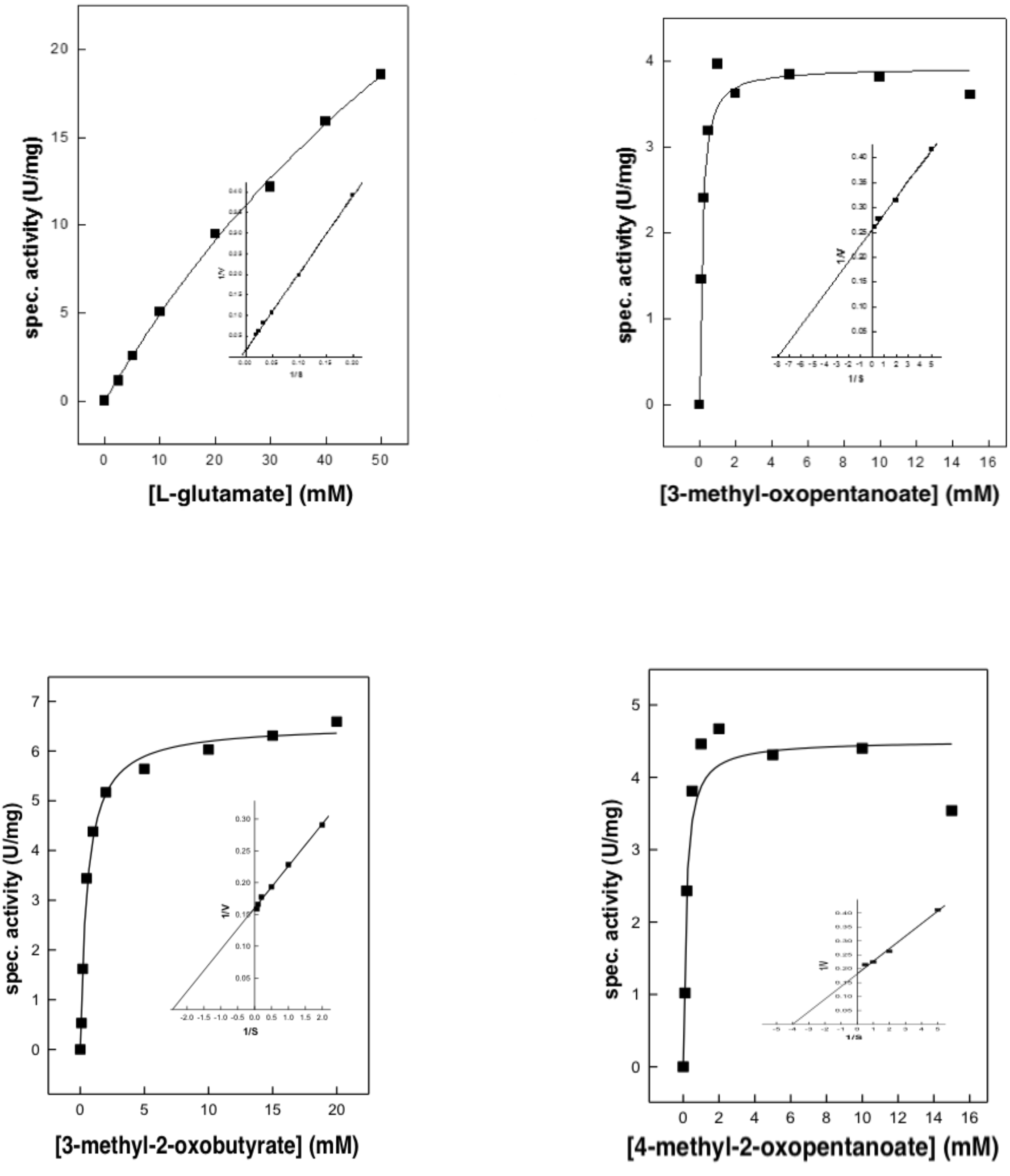

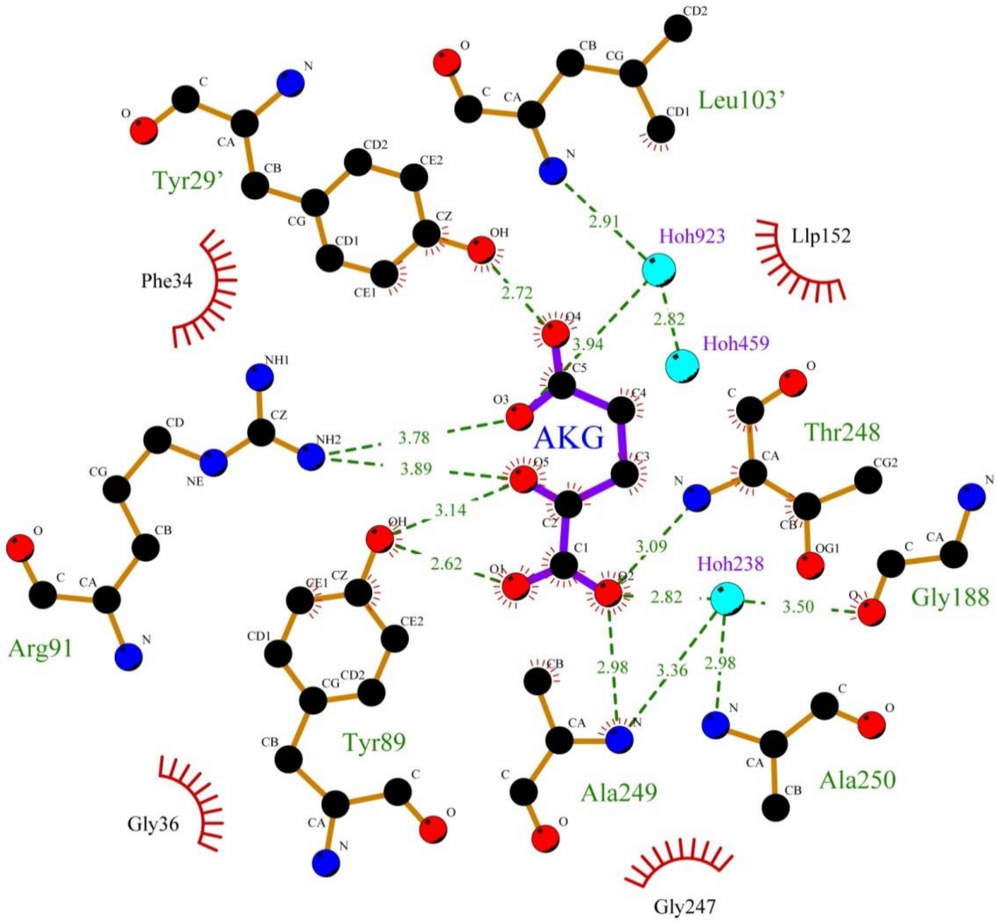

Figure S8. The contacts made of the α-ketoglutarate molecule as found in the GEO1900_AKG BCAT structure. Hydrogen bonds are depicted as green dashed lines and the corresponding distances are labelled. Residues of the adjacent subunit have apostrophe added to their label. Other residues, which do not participate in the hydrogen bond network but form the active site cavity are labelled in black. Water molecules are shown as cyan spheres and labelled. Figure was prepared with LIGPLOT (Wallace et al., 1996).

Wallace A. C., Laskowski R. A., Thornton J. M. (1996). LIGPLOT: a program to generate schematic diagrams of protein-ligand interactions. *Protein Eng.*, **8**, 127-134.

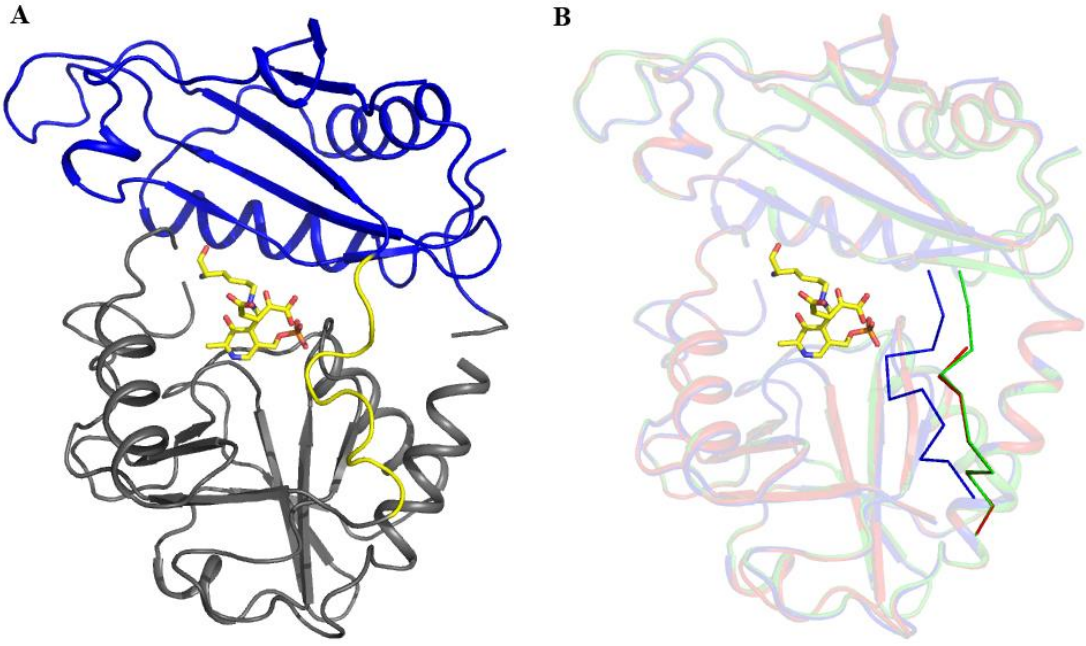

Figure S9. A cartoon representation of the GEO1900 subunit in the complex structure with α-ketoglutarate. The small domain is shown in blue, the large domain is shown in grey, and the interdomain loop is shown in yellow. The covalently bound PLP molecule is shown in yellow as well as the bound ligand. (B) Representation of the superimposed structures of the A subunit of GEO1900_AKG (blue), B subunit of GEO1900_AKG (green) and B subunit of GEO1900_holo (red). The interdomain loops are shown as ribbons. Other parts of the structures are shown transparently for clarity.

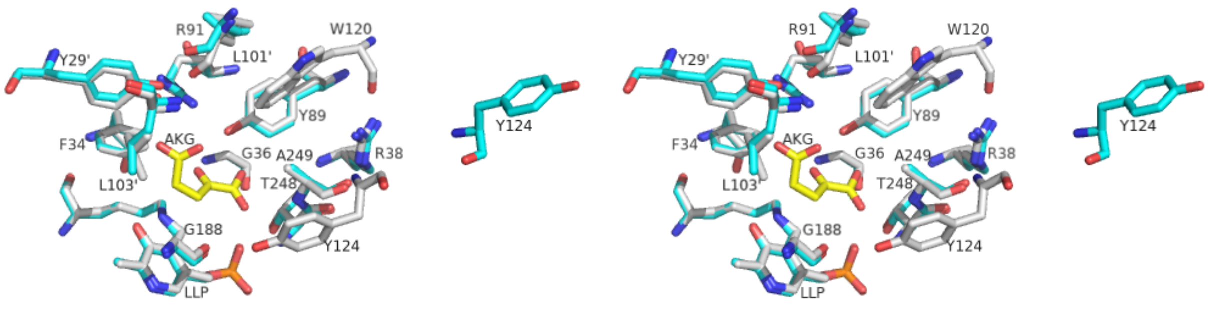

Figure S10. A stereo-view of the superposition of GEO1900 BCAT active site. Residues of the GEO1900_holo (B subunit) are shown in cyan and for GEO1900_AKG (A subunit) – in white. The substrate is shown in yellow.

Figure S11. The rate dependence of GEO1900 BCAT on the concentrations of pyruvate (▼), α-ketoglutarate (o).

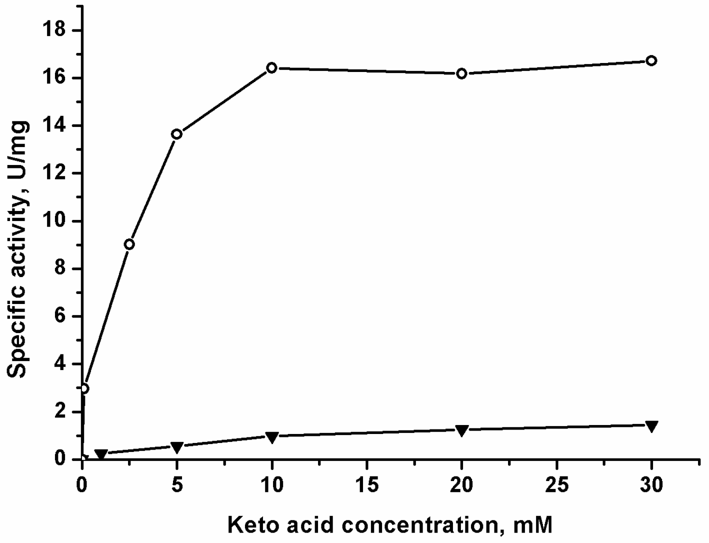

Supplement: Supplementary file 1 [file Data_Sheet_1.PDF]
